# Integrated tissue-specific flavoromics and metabolomics reveal species-specific aroma and radical scavenging chemotypes in *Dictyophora* mushrooms

**DOI:** 10.1016/j.fochx.2026.104632

**Published:** 2026-10-17

**Authors:** Mingzheng Duan, Chenggang Zhao, Qiuju Wu, Ming Tao, Xue Wang, Tingfen Lu, Tingting Qu, Min Li, Ting Li, Lihua Tang, Muhammad Junaid Rao, Zeping Wang, Xiaojian Wu

**Affiliations:** a Guangxi Academy of Agricultural Sciences, Nanning 530007, China; b Key Laboratory of Edible Fungi Resources Innovation Utilization and Cultivation, Zhaotong University, Zhaotong 657000, China; c College of General Education, Guangxi Vocational University Of Agriculture, Nanning 530009, China; d State Key Laboratory for Development and Utilization of Forest Food Resources, Zhejiang A&F University, Hangzhou 311300, China; e Zhejiang Key Laboratory of Non-wood Forest Products Quality Regulation and Processing Utilization, Zhejiang A&F University, Hangzhou 311300, China

**Keywords:** *Dictyophora* spp., Volatile organic compounds, Phenolic metabolites, Radical scavenging capacity, Flavoromics

## Abstract

*Dictyophora indusiata* and *D. rubrovalvata* exhibit distinct textural and sensory qualities, yet their chemotypes remain unresolved. Here, we integrated biochemical assays with metabolomics to compare caps and volvas across species. *D. indusiata* caps showed higher total phenolics (6.05 vs. 3.80 mg GAE/g DW), DPPH (2.45 vs. 0.74 μmol TE/g DW), and ABTS (4.85 vs. 3.15 μmol TE/g DW) than *D. rubrovalvata* caps. Volatile profiling suggested higher total volatiles in *D. indusiata* caps (339.2 vs. 160.6 μg/g FW), dominated by terpenoids, esters, ethers, and heterocyclics, whereas *D. rubrovalvata* caps had green-note C6 alcohols. Multivariate analyses suggest species-specific divergence in cap volatiles but relative conservation in volvas. Primary metabolites suggested contrasting carbon partitioning and organic acids between tissues and species. Collectively, these data define tissue-resolved chemotypes that provide a chemical basis for the predicted superior aroma complexity and in-vitro radical scavenging potential of *D. indusiata* caps, suggesting potential as high-value functional ingredients.

## Highlights

• Integrated flavoromics & metabolomics of two *Dictyophora* spp. in cap and volva.
• *D. indusiata* cap exhibits higher in-vitro radical scavenging, terpenoids & esters.
• *D. rubrovalvata* cap contains higher levels of sugars, glutamate & C6 alcohols.
• *D. indusiata* volva accumulates more malic acid; *D. rubrovalvata* volva has little.
• Species- and tissue-specific chemotypes inform functional food development.

## 1 Introduction

Edible mushrooms are increasingly recognized as multifunctional food resources that combine desirable sensory attributes with a dense matrix of bioactive compounds (K. Kumar et al., 2021). Beyond their characteristic “umami” taste and complex aromas, many culinary mushrooms provide substantial quantities of proteins, dietary fiber, vitamins, minerals, and diverse secondary metabolites, including phenolics and terpenoids, that contribute to antioxidant and anti-inflammatory activities (Das et al., 2021; K. Kumar et al., 2021). As consumers increasingly seek natural, minimally processed foods with added health benefits, mushrooms are being repositioned from simple vegetables to multifunctional ingredients capable of modulating both sensory quality and nutritional value (J. Yang et al., 2025). In particular, the demand for natural flavorings and clean-label antioxidants to replace synthetic additives has intensified interest in mushroom-derived volatiles and phenolic compounds as potential functional ingredients for soups, sauces, seasonings, and ready-to-eat products(Michalska et al., 2025). Therefore, understanding mushroom flavor and bioactivity is a food chemistry priority for selecting species/tissues that maximize sensory appeal and efficacy (Kumar et al., 2021). However, chemical profiling alone does not fully predict human odor perception, as matrix effects can significantly modulate sensory outcomes; thus, integrating chemical analysis with sensory evaluation is essential for comprehensive flavor characterization. Within this broader context, the genus *Dictyophora* provides a useful model because its culinary and medicinal value depends on tissue-specific chemistry that remains poorly resolved.

The genus *Dictyophora* (bamboo fungus or veiled lady) is a highly valued edible mushroom in East and Southeast Asia. Among them, *Dictyophora indusiata* and *Dictyophora rubrovalvata* are prized in Chinese, Japanese, and Southeast Asian cuisines for their unique morphology, delicate texture, and reputed medicinal properties, including immunomodulatory, hypolipidemic, and antioxidant effects attributed largely to polysaccharides and phenolic constituents (Kumar & Xu, 2025; Li et al., 2025). *D. indusiata*, commonly known as bamboo fungus or “zhu sheng,” is characterized by a conspicuous lace-like indusium and is widely used in soups and hotpots, where its crisp yet fragile texture and distinctive aroma are highly valued (Kumar & Xu, 2025). In contrast, *D. rubrovalvata* is characterized by a reddish volva and a relatively compact, spongy stipe. Its fresh fruiting body has extremely high moisture content (approximately 85–90%) (Meng et al., 2023; Zheng et al., 2024), making it highly perishable after harvest. While it is commonly dried for commercial distribution, its dried product is prone to breakage and browning during long-term storage, which poses challenges for maintaining commercial quality (Li et al., 2025; Meng et al., 2023; Zheng et al., 2024). Despite their increasing economic importance and clear differences in culinary performance, systematic comparative studies of these two species remain scarce. Existing work has largely focused on polysaccharide structure and general antioxidant capacity at the whole-fruiting-body level (Kumar & Xu, 2025; Li et al., 2025), leaving unresolved how species-specific metabolite profiles underpin their distinct flavor, aroma, and functional attributes, hindering rational selection for product development.

The *Dictyophora* fruiting body is structurally heterogeneous, comprising a cap, a stipe, an indusium, and a volva (Xie et al., 2025). Culinary practice indicates that the cap carries the characteristic flavor, while the volva contributes more to mouthfeel, a specialization likely associated with differential allocation of primary metabolites (sugars, organic acids, glutamic acid) and secondary metabolites (phenolics, terpenoids, esters), as well as distinct volatile organic compound (VOC) profiles (Das et al., 2021). In *D. indusiata*, the cap is consistently reported to emit a more intense and pleasant aroma than either its own volva or the cap of *D. rubrovalvata*. However, most previous studies have homogenized whole fruiting bodies or focused on dried products (Burapapadh & Saokham, 2021; Wang et al., 2021), thereby averaging out structural complexity and obscuring tissue-specific chemotypes associated with sensory perception. This lack of tissue-resolved information hampers our understanding of how structural differentiation translates into sensory and nutritional divergence. To link this structural heterogeneity to sensory and functional outcomes, it is necessary to integrate non-volatile taste compounds with volatile aroma-active molecules.

In food chemistry, flavor perception integrates non-volatile taste compounds and volatile aroma molecules. Sugars contribute sweetness and modulate Maillard reactions; organic acids (citric, malic) influence sourness and buffering capacity; and free amino acids, especially glutamic acid, drive umami, synergizing with nucleotides to enhance savory notes (J. Wang et al., 2020; Xie et al., 2025). Concurrently, volatile organic compounds (VOCs) such as terpenoids, esters, alcohols, ketones, heterocyclics, and sulfur compounds, define mushroom odor, but only those with low odor thresholds exert disproportionate sensory impact, making relative odor activity value (rOAV) analysis essential to identify key aroma contributors (Deng et al., 2024). Phenolic compounds add another critical dimension linking food chemistry to human health (Guo et al., 2026; Rao & Zheng, 2025; Yuan et al., 2026): mushroom phenolics (phenolic acids, flavonoids, derivatives) are potent radical scavengers and metal chelators that mitigate oxidative stress, with higher phenolic content associated with enhanced radical scavenging capacity and chronic disease protection (Abdelshafy et al., 2022; Duan, Chang, He, et al., 2026; Rao et al., 2026). Similar radical scavenging mechanisms have been reported in other plants under stress (Duan, Chang, Liang, et al., 2026; Duan, Lu, Wang, et al., 2026; Zhang et al., 2025). For *Dictyophora* species, however, no integrated flavoromics combining taste-active metabolites, rOAV-weighted VOCs, and tissue-resolved phenolic profiles has been reported, and the interplay between phenolic and volatile pathways, both derived from shared phenylpropanoid and isoprenoid precursors, remains unexplored. Consequently, the molecular basis for the perceived differences between *D. indusiata* (superior aroma, high antioxidant activity) and *D. rubrovalvata* (different texture, storage properties) is largely speculative. Key gaps include absence of comparative phenolic metabolomics distinguishing cap and volva; lack of rOAV-based volatile profiling at the tissue level; and no systematic multivariate analysis linking primary metabolites (sugars, organic acids, glutamate) to volatile and phenolic repertoires. Addressing these gaps is essential for evidence-based valorization, guiding cap use for aroma-rich applications and volvas for texture or health formulations.

The present study was designed to address these gaps by integrating tissue-specific flavoromics and metabolomics of *D. indusiata* and *D. rubrovalvata*. Building on the hypothesis that the cap of *D. indusiata* represents a distinct chemotype optimized for aroma and antioxidant function, we combined biochemical assays, comprehensive volatile and phenolic profiling, to characterize cap and volva chemistry. Specifically, this study aims to: (i) reveal how primary metabolites, volatiles, and phenolics are differentially allocated between cap and volva; (ii) identify species-specific discriminant markers that underpin differences in aroma and in-vitro radical scavenging potential; and (iii) provide a food-chemistry rationale for the preferential use of *D. indusiata* cap as a high-value functional ingredient. These findings are expected to deepen understanding of structure-function relationships in edible mushrooms and support the development of *Dictyophora*-based products with optimized sensory quality and health benefits.

## 2 Materials and methods

### 2.1 Mushroom materials, cultivation, and sample collection

*Dictyophora indusiata* and *D. rubrovalvata* strains were provided by the Guangxi Academy of Agricultural Sciences. Cultivation was conducted at the experimental base of Zhaotong University (Yunnan, China) starting in late March 2025. Trenches were prepared, filled with uncomposted bamboo sawdust, and covered with soil. Each strain was fragmented into chestnut-sized pieces, placed uniformly on the soil surface, and overlaid with approximately 5 cm of soil. Rice straw mulch was applied to maintain moisture. Small arched shelters covered with shade nets were erected to prevent mycelial scorching.

During the peak production period (late August 2025), mature fruiting bodies were collected. Tissues were dissected into cap and volva for analysis. All samples were immediately flash-frozen in liquid nitrogen and stored at −80 °C until further analysis. All assays were performed in triplicate (*n* = 3 biological replicates) for each tissue type and species.

### 2.2 Determination of sugars, starch, and organic acids

Glucose, soluble sugar, sucrose, starch, citric acid, and malic acid were quantified using commercial assay kits (Suzhou Grace Biotechnology Co., China) according to the manufacturers' instructions. All absorbance measurements were performed on a microplate reader (SpectraMax 190, Molecular Devices, San Jose, CA, USA).

For soluble sugar (G0501W48), sucrose (G0506W), and reducing sugar (G0502W), lyophilized sample (0.1 g) was extracted with 1.5 mL of 80% ethanol at 50 °C for 20 min, centrifuged (12,000 rpm, 10 min), and the supernatant used. Soluble sugar: 25 μL diluted extract +30 μL working solution (Reagent 1 in Reagent 2) + 250 μL H₂SO₄, heated at 95–100 °C for 10 min, read at 620 nm. Sucrose: 40 μL extract +20 μL Reagent 1 + 360 μL working solution, heated similarly, read at 620 nm (0.3 mg/mL sucrose standard). Reducing sugar: 100 μL extract +100 μL DNS reagent (Reagent 1), heated at 95 °C for 10 min, cooled, diluted with 1000 μL water, read at 500 nm (glucose standard 0–1 mg/mL). For glucose (G0504W), sample (0.1 g) was extracted with 1 mL distilled water, centrifuged, and 10 μL supernatant mixed with 20 μL Reagent 1 and 170 μL Reagent 2 mix (Reagent 2 A:2B = 2:1), incubated at 37 °C for 30 min in the dark, read at 520 nm (1 mg/mL glucose standard). All results expressed as mg/g dry weight (mg/g DW).

Starch was quantified using an acid hydrolysis-anthrone kit (G0507W). The sample (0.05 g) was extracted with 1 mL Reagent 1 at 50 °C for 30 min, centrifuged, and the pellet was suspended in 0.5 mL distilled water, gelatinized at 95 °C for 15 min, then hydrolyzed with 0.35 mL concentrated HCl for 15 min. After adding 0.85 mL water and centrifugation, the supernatant (50 μL) was mixed with 250 μL working solution (Reagent 2 dissolved in H₂SO₄), heated at 95 °C for 10 min, and absorbance read at 620 nm. The result was multiplied by 0.9 to convert glucose to starch.

For citric acid (G0864W) and malic acid (G0862W48) (0.1 g) of sample was homogenized in 1 mL extraction buffer on ice, centrifuged (12,000 *g* or rpm, 10 min, 4 °C), and the supernatant used. For citric acid, 10 μL supernatant was mixed with 10 μL Reagent 1, 40 μL Reagent 2, and 40 μL extraction buffer, incubated at 25 °C for 20 min, and absorbance read at 470 nm (citric acid standard curve 0–1 mg/mL). For malic acid, 20 μL supernatant was mixed with 10 μL each of Reagents 1–4, 130 μL Reagent 5, and 10 μL Reagent 6, incubated at 37 °C for 30 min in the dark, and absorbance read at 450 nm (malic acid standard 0.2 μmol/mL). Results were expressed as mg/g dry weight (mg/g DW) based on manufacturer's standard curves.

### 2.3 Determination of glutamic acid

Glutamic acid was quantified using a commercial kit (G0416W, Suzhou Grace Biotechnology Co., China) following the microplate method. Lyophilized cap or volva tissue (0.1 g) was extracted with 1 mL of the supplied extraction buffer, then 1 μL of Reagent 1 was added, incubated for 5 min, and centrifuged at 12,000 rpm for 10 min. The supernatant (20 μL) was mixed with Reagent 2 (20 μL), Reagent 3 (10 μL), and Reagent 4 (10 μL), and incubated at 30 °C for 20 min in the dark. Absorbance was read at 450 nm using a microplate reader (SpectraMax 190, Molecular Devices). A sample blank (extraction buffer instead of Reagent 4) was used for each sample. Glutamic acid content was calculated using a standard curve (y = 0.0829× – 0.0098) and expressed as mg/g dry weight (converted from nmol/g using molecular weight 147.13).

### 2.4 Determination of total phenolic content

Total phenolic content (TPC) was measured with a commercial kit (G0117W, Suzhou Grace Biotechnology) based on the Folin-Ciocalteu method. Lyophilized sample (0.03 g) was extracted with 1.5 mL of 60% ethanol at 60 °C for 2 h with shaking, then centrifuged at 12,000 rpm for 10 min. The supernatant (10 μL) was mixed with Reagent 1 (50 μL) and incubated for 3 min at 25 °C in the dark. Then Reagent 2 (50 μL) and distilled water (90 μL) were added, and the mixture was incubated for another 30 min. Absorbance was measured at 760 nm (SpectraMax 190). A blank (water instead of sample) was run. TPC was calculated from the standard curve (y = 0.3494× – 0.0006, where x = μg gallic acid) and expressed as mg gallic acid equivalent per gram dry weight (mg GAE/g DW).

### 2.5 Determination of radical scavenging capacities

#### 2.5.1 DPPH• radical scavenging capacity

The DPPH assay was performed using a commercial kit (G0128W48, Suzhou Grace Biotechnology). Lyophilized sample (0.05 g) was extracted with 1 mL of 80% methanol under ultrasound (60 °C, 200–300 W, 30 min, with intermittent shaking), then centrifuged at 12,000 rpm for 10 min. The supernatant (150 μL) was mixed with 150 μL of DPPH working solution (prepared as per kit instructions) and incubated at 25 °C in the dark for 30 min. After centrifugation (12,000 rpm, 5 min), 200 μL of the mixture was transferred to a 96-well plate, and absorbance was read at 517 nm (SpectraMax 190). A sample blank (80% methanol instead of DPPH solution) and a reagent blank (80% methanol instead of sample) were included. The scavenging rate (%) was calculated, and the capacity was expressed as μmol Trolox equivalent per gram dry weight (μmol TE/g DW) using a Trolox standard curve (y = 2.8486× + 0.7084, where x = μg Trolox/mL).

#### 2.5.2 ABTS• + radical scavenging capacity and rate

The ABTS assay was carried out with a commercial kit (G0127W, Suzhou Grace Biotechnology). Sample extraction followed the same procedure as for DPPH (Section 2.5.1). The ABTS working solution was prepared by mixing equal volumes of Reagent 1 (ABTS solution) and Reagent 2 (potassium persulfate), allowing the mixture to react for 12 h in the dark, then diluting with ethanol to an absorbance of 0.70 ± 0.02 at 734 nm. For the assay, 10 μL of sample extract was mixed with 190 μL of ABTS working solution and incubated at 25 °C for 6 min in the dark. Absorbance was measured at 734 nm. A sample blank (80% methanol instead of ABTS working solution) and a reagent blank (80% methanol instead of sample) were used. The scavenging rate (%) was calculated, and the capacity was expressed as μg Trolox equivalent per gram dry weight (μg TE/g DW) using a Trolox standard curve (y = 0.5042× – 1.4213, where x = μg Trolox/mL).

### 2.6 Volatile organic compound analysis by GC–MS

#### 2.6.1 Sample preparation and SPME extraction

For each biological replicate (cap and volva of both *D. indusiata* and *D. rubrovalvata*), 500 mg of frozen tissue was weighed and transferred into a 20 mL headspace vial (Agilent, Palo Alto, CA, USA) containing 2 mL of saturated sodium chloride solution to suppress enzymatic reactions and enhance volatile partitioning. Vials were immediately sealed with crimp-top caps fitted with TFE‑silicone septa (Agilent). Each sample was spiked with 20 μL of internal standard solution (10 μg/mL 3-hexanone-d5 in methanol, Sigma-Aldrich, St. Louis, MO, USA) prior to extraction. Samples were equilibrated at 60 °C for 5 min under agitation. Volatile organic compounds (VOCs) were extracted using a 120 μm DVB/CWR/PDMS Solid-Phase Microextraction (SPME) Arrow fiber (Agilent), which was exposed to the sample headspace for 15 min at 60 °C. After extraction, the fiber was retracted and immediately inserted into the GC injection port for thermal desorption. Volatile contents are expressed on a fresh weight (FW) basis. The moisture content of fresh *D. indusiata* and *D. rubrovalvata* tissues was not directly measured in this study; however, typical values for fresh *Dictyophora* fruiting bodies have been reported at approximately 85–90% (Meng et al., 2023; Zheng et al., 2024). For stoichiometric conversion to dry weight basis, these literature values should be applied with caution. All comparative analyses were performed on the same weight basis (FW vs. FW), ensuring internal consistency. The FW basis was chosen to reflect volatiles released from fresh tissue, which is directly relevant to consumer perception.

#### 2.6.2 GC–MS conditions

Desorption of VOCs from the SPME fiber was performed in the injection port of an Agilent 8890 gas chromatograph at 250 °C for 5 min in splitless mode. Separation was achieved on a DB-5MS capillary column (30 m × 0.25 mm i.d., 0.25 μm film thickness; 5% phenyl-polymethylsiloxane; Agilent). Helium (purity ≥99.99%) was used as carrier gas at a constant flow rate of 1.2 mL/min. The oven temperature program was as follows: initial temperature 40 °C held for 3.5 min, ramped at 10 °C/min to 100 °C, then at 7 °C/min to 180 °C, and finally at 25 °C/min to 280 °C with a 5 min hold. The transfer line temperature was maintained at 280 °C. Mass spectrometry was performed using an Agilent 7000D triple quadrupole mass spectrometer operating in electron impact (EI) mode at 70 eV. The ion source and quadrupole temperatures were set to 230 °C and 150 °C, respectively. Data acquisition was performed in full scan mode (*m*/*z* 35–500) for compound identification, and selected ion monitoring (SIM) was used for quantification. Compounds were identified by comparison of mass spectra with the NIST 20 library and by retention indices relative to a series of n-alkanes (C8–C40). Relative quantification was performed using 3-hexanone-d5 as the internal standard, with compound peak areas normalized to the internal standard peak area and expressed as μg/g fresh weight. Volatile compounds with relative odor activity value (rOAV) ≥ 1 were considered aroma-active contributors (Fang et al., 2024).

Relative quantification was performed using the internal standard method according to the following equation:

$$X_{i}=\frac{V_{s}\times C_{s}}{M}\times \frac{I_{i}}{I_{s}}\times 10^{-3}$$

where $X_{i}$ is the content of compound i (μg/g fresh weight), $V_{s}$ is the volume of internal standard added (μL), $C_{s}$ is the concentration of the internal standard (μg/mL), M is the sample mass (g), $I_{s}$ is the peak area of the internal standard, and $I_{i}$ is the peak area of compound i.

Whereas the rOAV was calculated for each volatile compound to assess its contribution to overall aroma, using the formula:

rOAVi=Ci/Ti

where $C_{i}$ is the relative content (μg/g) and $T_{i}$ is the odor detection threshold (μg/g). The odor threshold values used for rOAV calculation were obtained from the compilation by van Gemert (Van Gemert, 2011). Odor descriptions for volatile compounds were sourced from publicly available flavor databases (The Good Scents Company; http://www.thegoodscentscompany.com/, Perflavory; http://perflavory.com/, Odor; http://www.odour.org.uk/, and Food Flavor Lab; http://foodflavorlab.cn/) and published literature (Huang et al., 2022; Xue et al., 2022). rOAV analysis was performed to predict the contribution of each volatile compound to the overall aroma profile. The raw intensities and odor thresholds for all detected volatile compounds are provided in Supplementary Table S1.

### 2.7 LC-MS/MS analysis of phenolic metabolites

#### 2.7.1 Sample extraction

Fresh mushroom tissues (cap and volva of both species) were frozen in liquid nitrogen and lyophilized using a freeze dryer (Scientz-100F) for 63 h. The dried samples were ground to a fine powder using a ball mill (MM 400, Retsch) at 30 Hz for 1.5 min. Approximately 30 mg of powder was weighed into a 2 mL tube, and 1500 μL of pre-cooled (−20 °C) 70% (*v*/v) methanol containing 250 μg/mL of an internal standard (prepared from a 1000 μg/mL stock solution diluted with 70% methanol) was added. The mixture was vortexed for 30 s every 30 min (6 times total), then centrifuged at 12,000 rpm for 3 min. The supernatant was filtered through a 0.22 μm membrane filter and transferred to an autosampler vial for ultra-performance liquid chromatography tandem mass spectrometry (UPLC-MS/MS) analysis (W. Chen et al., 2013). Although a single-solvent system (70% methanol) was used, this solvent has been widely validated for polyphenolic extraction from mushrooms (An et al., 2020). We acknowledge that some highly lipophilic or strongly bound phenolic conjugates may be under-extracted; however, the detection of a diverse range of phenolic acids and phenylpropanoids suggests good coverage.

#### 2.7.2 UPLC conditions

Chromatographic separation was performed on an ExionLC™ AD system (SCIEX) equipped with an Agilent SB-C18 column (1.8 μm, 2.1 mm × 100 mm). The mobile phase consisted of (A) water with 0.1% formic acid and (B) acetonitrile with 0.1% formic acid. The gradient program was: 0–9.0 min, 5–95% B; 9.0–10.0 min, hold at 95% B; 10.0–11.1 min, return to 5% B; and 11.1–14.0 min, equilibrate at 5% B. The flow rate was 0.35 mL/min, column temperature 40 °C, and injection volume 2 μL.

#### 2.7.3 Mass spectrometry conditions

Mass spectrometry was performed on a QTRAP 6500+ system (SCIEX) equipped with an electrospray ionization (ESI) source. The source parameters were: temperature 500 °C; ion spray voltage +5500 V (positive mode) / –4500 V (negative mode); ion source gas 1 and gas 2 at 50 and 60 psi, respectively; curtain gas at 25 psi; collision-activated dissociation (CAD) set to high. Data acquisition was carried out in multiple reaction monitoring (MRM) mode, with collision gas (nitrogen) set to medium. Declustering potential (DP) and collision energy (CE) were optimized for each MRM transition. A specific set of MRM transitions was monitored for each period according to the elution time of the metabolites. Peak integration and correction were performed using Analyst 1.6.3 software. Metabolites were identified by comparing their MRM transitions, retention times, and fragmentation patterns with the in-house Metware Database (MWDB). The level of identification (Level 1–3) was assigned according to the Metabolomics Standards Initiative (MSI) criteria: Level 1 (authentic standard comparison), Level 2 (putative identification based on spectral matching), and Level 3 (tentative identification based on class-specific spectral features) (W. Chen et al., 2013; Sumner et al., 2007). The identification parameters of all detected phenolic metabolites, including compound identifiers, mass spectrometric transitions, MSI level, and database mapping information, are provided in Supplementary Table S2. Quantification was based on peak area normalization relative to the internal standard (2-chlorophenylalanine; 1PPM mg/L).

### 2.8 Orthogonal partial least-squares discriminant analysis (OPLS-DA)

OPLS-DA was performed using the *MetaboAnalystR* package (version 1.0.1) to maximize the separation between groups and identify discriminant metabolites (Thévenot et al., 2015). The model quality was evaluated by R^2^Y (cumulative explained variation of the Y-matrix) and Q^2^ (cumulative predictive ability), with Q^2^ > 0.5 considered acceptable and Q^2^ > 0.9 considered excellent. Model validity was assessed by permutation testing (200 random permutations, *p* < 0.05), where the intercepts of R^2^Y and Q^2^ were examined to exclude overfitting (Eriksson et al., 2013). Variable importance in projection (VIP) scores were extracted from the OPLS-DA model; metabolites with VIP > 1.0 were considered potential discriminant markers. VIP scores for all detected volatile compounds are provided in Supplementary Table S3 (full compound names are listed in Supplementary Table S1). S-plots were generated to visualize the covariance (p[1]) and correlation (p(corr)[1]) of each metabolite, highlighting those with both high magnitude and high reliability for group separation.

### 2.9 Differential metabolite selection and correlation analysis

For two-group comparisons (e.g., Ccap vs. Hcap, Cvol vs. Hvol), differential volatile compounds were identified based on a combination of VIP > 1.0 and absolute log₂ fold change (|log₂FC|) ≥ 1.0. All statistical tests were performed using R (version 4.2.0). Pearson's correlation coefficients among biological replicates were calculated to assess intra-group consistency and analytical reproducibility. The correlation matrix was visualized as a heatmap using the *ComplexHeatmap* package (version 2.12.0), with a color scale ranging from −1 to 1. For flavor network analysis, correlations with ∣r∣>0.8 and p<0.05 were retained, and networks were visualized using the *igraph* package in R. Overlaps and unique volatile compounds among different pairwise comparisons were visualized using Venn diagrams generated via the EVenn web-based platform (https://www.bic.ac.cn/EVenn/#/).

### 2.10 Multivariate statistical analysis

Multivariate analyses were conducted to visualize sample relationships and identify discriminant metabolites. Hierarchical Clustering Analysis (HCA), principal Component Analysis (PCA), and K-means were performed using the *ComplexHeatmap* package (version 4.1.2) in R to visualize the global metabolite profiles (flavoromics and phenolics) and to assess sample grouping patterns (Chong & Xia, 2018). The resulting heatmap was color-scaled to represent normalized metabolite abundances whereas in PCA the marginal boxplots were incorporated to display the distribution of PC1 and PC2 scores across groups, with statistical significance evaluated by Student's t-test for pairwise comparisons (Chanana et al., 2020). The radar chart was developed in R (fmsb) version 0.7.1.

Biochemical parameters including glucose, reducing sugar, soluble sugar, sucrose, starch, citric acid, malic acid, and glutamic acid were quantified as described in 2.2, 2.3. All data are presented as mean ± standard deviation (SD) from three biological replicates (n=3). Statistical comparisons between two groups (e.g., cap vs. volva within species, or species within tissue) were performed using Student's t-test. For multiple comparisons, Duncan's multiple range test was applied, with significance defined at p<0.05.

## 3 Results

### 3.1 Tissue-specific distribution of total phenolics and radical scavenging capacities

Representative photographs of the cap and volva tissues from both species showed that *D. indusiata* (C) exhibited a characteristic brownish, delicate indusium (skirt) extending from the cap, consistent with its crisp texture and known autolytic properties (Fig. 1A). In contrast, *D. rubrovalvata* (H) displayed a more compact morphology with a reddish‑tinged volva and a spongy stipe. These distinct morphological traits align with the species-specific differences in texture and autolysis, which are core characteristics distinguishing the two *Dictyophora* resources. The radical scavenging capacities and total phenolic contents of cap and volva tissues are presented (Fig. 1B–E). Total phenolic content (Fig. 1B) was highest in *D. indusiata* cap (6.05 mg GAE/g DW), significantly exceeding that of *D. rubrovalvata* cap (3.80 mg GAE/g DW). A similar trend was observed in the volva, with *D. indusiata* (1.77 mg GAE/g DW) showing higher values than *D. rubrovalvata* (1.15 mg GAE/g DW).

**Fig. 1:**
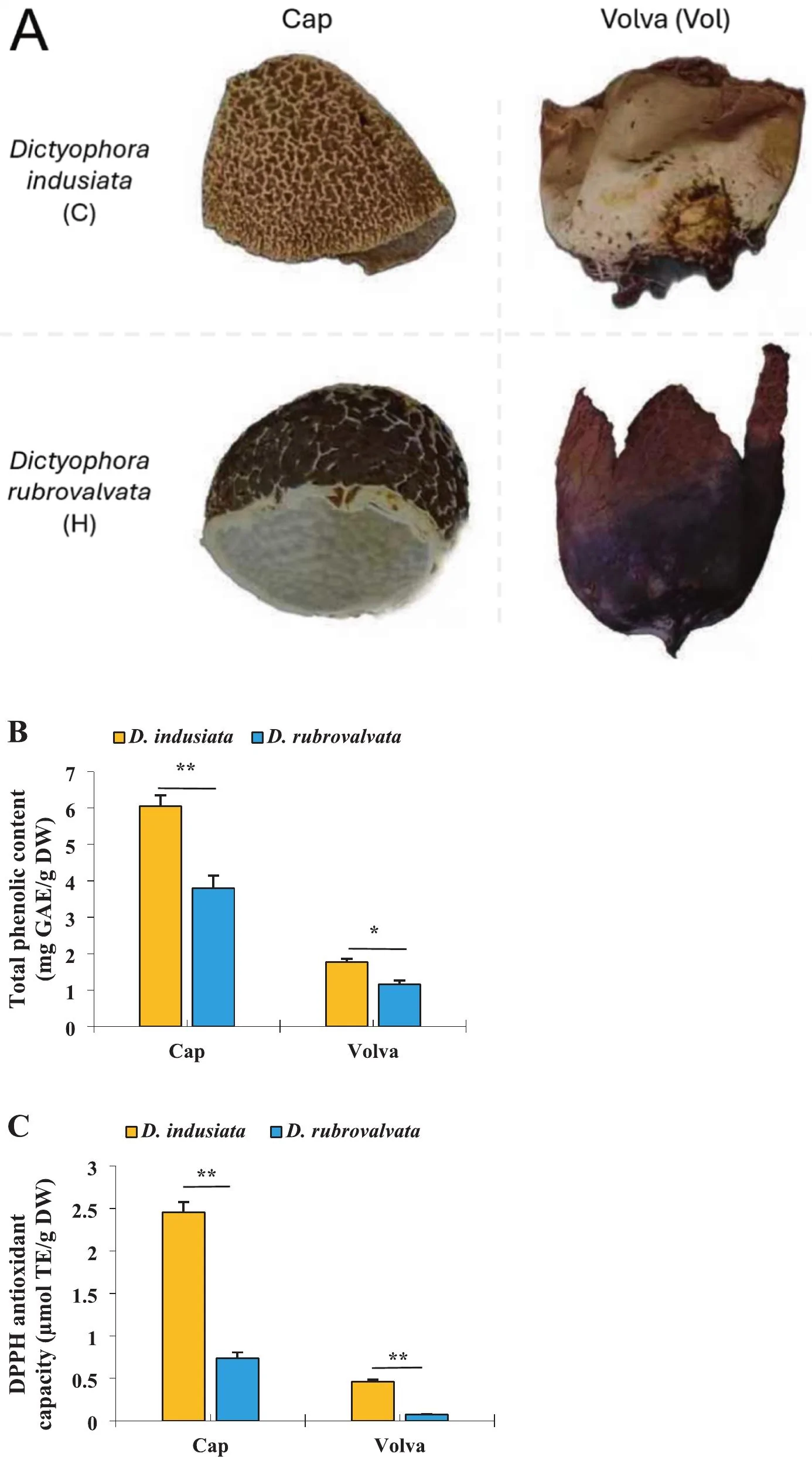

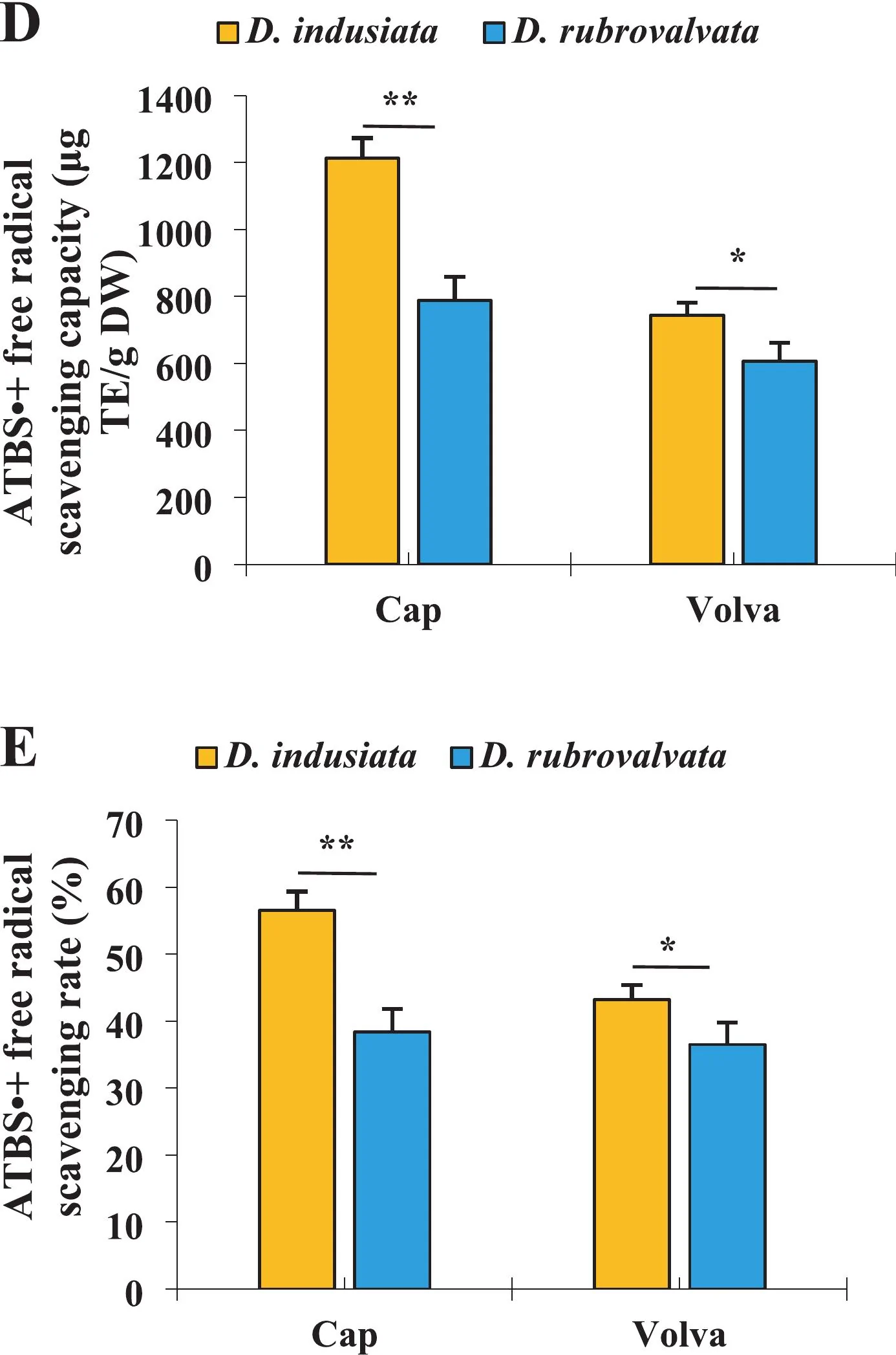
Phenotypic and radical scavenging potential of cap and volva tissues from *D. indusiata* (C) and *D. rubrovalvata* (H). (A) Representative photographs of cap and volva from both species. (B) Total phenolic content. (C) DPPH• radical scavenging capacity. (D) ABTS• + radical scavenging capacity. (E) ABTS• + radical scavenging rate (%). Data are presented as mean ± SD (*n* = 3). Significance levels: * p<0.05, ** p<0.01, (Student's t-test).

DPPH• radical scavenging capacity (Fig. 1C) was markedly higher in *D. indusiata* cap (2.45 μmol TE/g DW) compared to *D. rubrovalvata* cap (0.74 μmol TE/g DW). In the volva, *D. indusiata* (0.46 μmol TE/g DW) also exhibited significantly higher activity than *D. rubrovalvata* (0.07 μmol TE/g DW). ABTS• + radical scavenging capacity (Fig. 1D) followed a consistent pattern: *D. indusiata* cap (1212.7 μg TE/g DW) was significantly higher than *D. rubrovalvata* cap (788.0 μg TE/g DW), while the volva of *D. indusiata* (744.5 μg TE/g DW) also exceeded that of *D. rubrovalvata* (606.9 μg TE/g DW). The ABTS scavenging rate (Fig. 1E) was consistent with these findings, with *D. indusiata* cap showing the highest value (56.5%) among all samples. Collectively, these results suggest that *D. indusiata* cap possesses higher in-vitro radical scavenging potential compared to *D. rubrovalvata* cap, with the cap showing the most pronounced interspecies divergence. Given that the cap also serves as the primary source of the characteristic aroma in *D. indusiata*, subsequent analyses focused on comparing the volatile and phenolic profiles of caps to elucidate the molecular basis underlying these functional and sensory differences.

### 3.2 Carbohydrate, organic acid, and glutamic acid profiles in cap and volva

Significant differences in primary metabolites and taste-active compounds were observed between tissues and species (Fig. 2). In the cap, *D. rubrovalvata* (H) consistently exhibited markedly higher levels of glucose (38.7 mg/g DW), reducing sugar (86.9 mg/g DW), soluble sugar (54.6 mg/g DW), and starch (51 mg/g DW) than *D. indusiata* (C); however, C cap had slightly higher sucrose content (9.9 mg/g DW) than H cap (7.7 mg/g DW) (Fig. 2A–E), indicating a species-specific enrichment of readily available carbohydrates in the cap. Conversely, in the volva, C contained significantly higher glucose and soluble sugar than H, whereas these are nearly absent in the H volva (Fig. 2A and C). This reciprocal pattern suggests that *D. indusiata* preferentially allocates carbon reserves to the volva tissue, while *D. rubrovalvata* concentrates sugars in the cap.

**Fig. 2:**
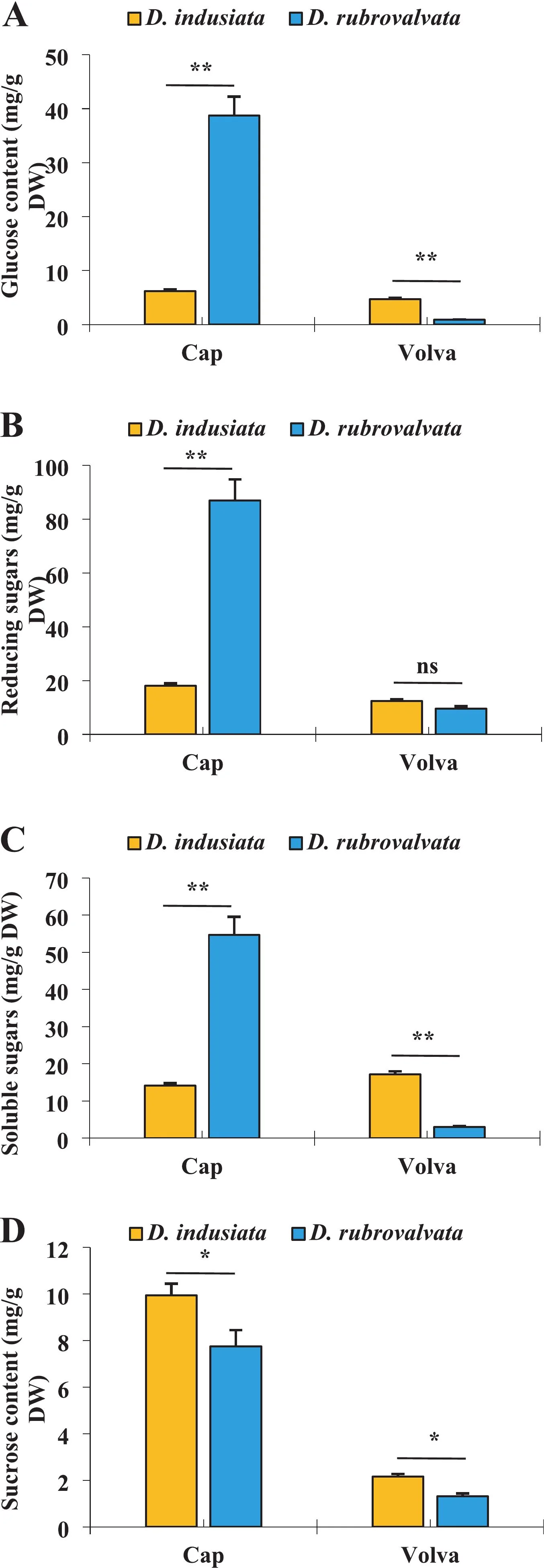

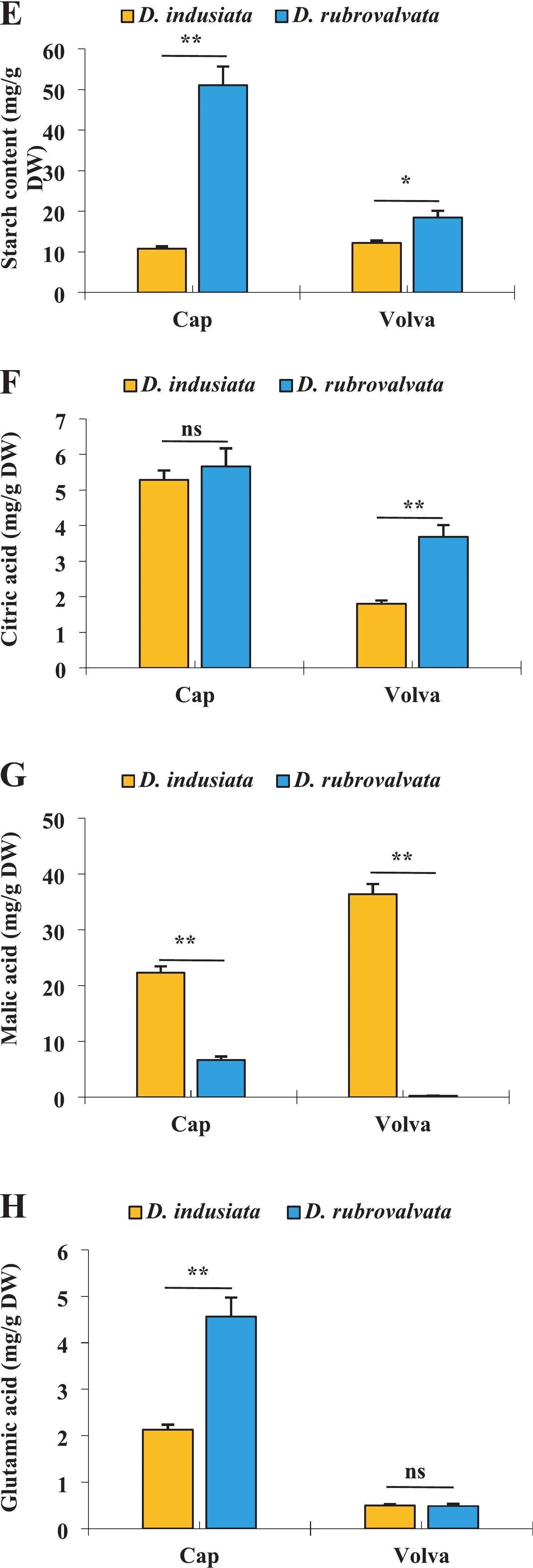
Biochemical composition of cap and volva tissues from *D. indusiata* and *D. rubrovalvata*. (A) Glucose, (B) reducing sugar, (C) soluble sugar, (D) sucrose, (E) starch, (F) citric acid, (G) malic acid, (H) glutamic acid. Data are presented as mean ± SD (*n* = 3). Significance levels: * p<0.05, ** p<0.01, ns = not significant (Student's t-test).

Organic acids showed contrasting trends. Citric acid was similar between species in the cap, but significantly higher in the H volva (3.68 mg/g DW) compared to C volva (1.8 mg/g DW) (Fig. 2F). Malic acid exhibited a striking reversal: C volva contained the highest concentration (36.39 mg/g DW) among all samples, while H volva (0.24 mg/g DW) had the lowest (Fig. 2G), implying species- and tissue-specific regulation of the TCA cycle intermediate pool.

Glutamic acid, a key umami contributor, was highest in the caps of both species, with H cap (4.56 mg/g DW) showing the greatest content than C cap (2.12 mg/g DW) (Fig. 2H). Volva tissues contained comparably low levels regardless of species. The glutamic acid content in *D. rubrovalvata* cap was within the range previously reported for *D. rubrovolvata* fruiting bodies (1.34–4.86 mg/g) (Liang et al., 2020), while *D. indusiata* cap (2.13 mg/g DW) was higher than some earlier whole-fruiting-body reports (0.54 mg/g) (Mau et al., 2001), likely reflecting tissue-specific enrichment in the cap. A recent study on *D. rubrovalvata* also confirmed that glutamic acid was the most abundant free amino acid across different tissues (7.44–11.52 mg/g total free amino acids), contributing to umami and sweetness (Wang, Han, et al., 2024). These comparisons support the conclusion that the cap, particularly in *D. rubrovalvata*, is a tissue specialized for umami-related metabolite accumulation.

### 3.3 Volatile organic compound profiles

Quantified relative contents (μg/g FW) of all detected volatile organic compounds are presented in Supplementary Table S4. Total volatile content varied significantly by tissue and species (Fig. 3A). *D. indusiata* cap exhibited the highest total volatiles (339.2 μg/g FW), nearly double that of *D. rubrovalvata* cap (160.6 μg/g FW). In the volva, *D. indusiata* also contained higher volatiles than *D. rubrovalvata*, however, the difference was less pronounced.

**Fig. 3:**
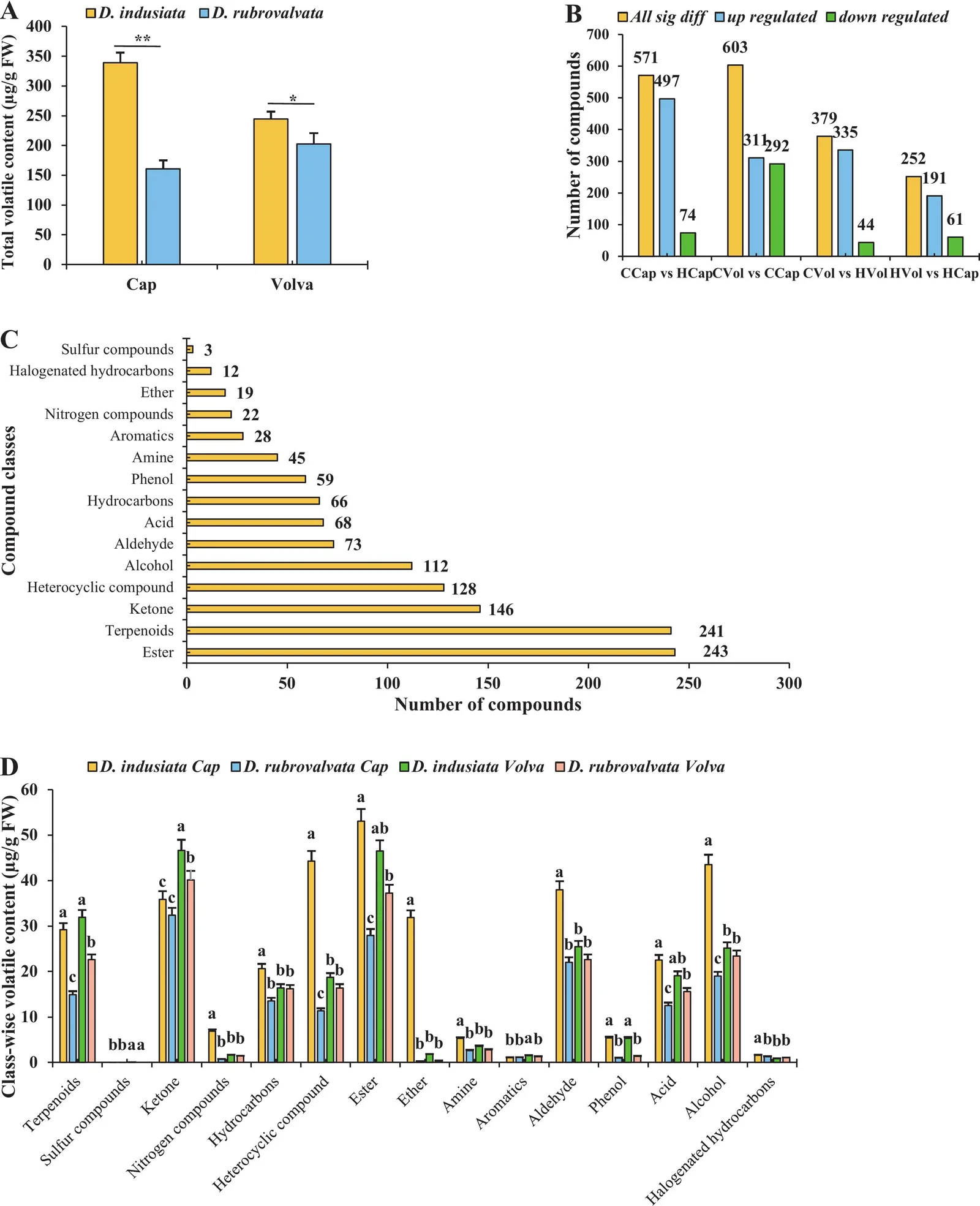
Volatile profiles of cap and volva tissues from *D. indusiata* (C) and *D. rubrovalvata* (H). (A) Total volatile content (μg/g FW). Data are presented as mean ± SD (*n* = 3). Significance levels: * p<0.05, ** p<0.01 (Student's t-test). (B) Number of differentially accumulated volatile compounds between tissue and species comparisons. (C) Number of volatile compounds classified by chemical class. (D) Class-wise volatile content (μg/g FW). Different lowercase letters (a, b, c) within each chemical class indicate significant differences among the four samples (p<0.05, Duncan's multiple range test).

Differential accumulation analysis suggested that the cap-to-cap comparison (Ccap vs. HCap) yielded the highest number of differentially accumulated compounds (571 total; 497 up-regulated in *D. indusiata*), indicating substantial species-level divergence in volatile metabolism (Fig. 3B). In contrast, the volva-to-volva comparison (Cvol vs. Hvol) showed fewer differences (379 total), suggesting greater metabolic conservation in the volva tissue. Chemical class distribution (Fig. 3C) showed that esters (243 compounds) and terpenoids (241 compounds) were the most abundant classes by compound count, followed by ketones (146) and heterocyclic compounds (128). Sulfur compounds, despite their potent aroma impact, were represented by only 3 compounds.

Class-wise quantification revealed striking tissue- and species-specific patterns (Fig. 3D). *D. indusiata* cap accumulated significantly higher levels of esters (53.1 μg/g FW), terpenoids (29.2 μg/g FW), alcohols (43.5 μg/g FW), and heterocyclic compounds (44.3 μg/g FW) compared to *D. rubrovalvata* cap (all p<0.05). Notably, ethers were abundant in *D. indusiata* cap (31.9 μg/g FW) but nearly absent in *D. rubrovalvata* cap (0.26 μg/g FW), representing the most dramatic class-wise difference between species. In the volva, *D. indusiata* retained higher terpenoid (31.9 μg/g FW) and ester (46.5 μg/g FW) levels, whereas *D. rubrovalvata* volva showed consistently lower values across most classes.

These data suggest that *D. indusiata* possesses a more diverse and quantitatively richer volatile repertoire, particularly in the cap, associated with elevated ester, terpenoid, and ether biosynthesis. The marked depletion of ethers and heterocyclics in *D. rubrovalvata* suggests fundamental differences in the biosynthetic capacity for aroma-active compounds between the two species. Collectively, the volatile profiles predict that *D. indusiata* will have a more complex and potentially more intense aroma, with the cap being the primary contributor to species-specific olfactory characteristics.

### 3.4 Multivariate analysis and comparative volatile profiles

Hierarchical clustering (Fig. 4A) grouped the four samples into three distinct clusters: the cap and volva of *D. rubrovalvata* (H) formed a single cluster, whereas the cap and volva of *D. indusiata* (C) each occupied separate clusters. This topology indicates that tissue type alone does not fully explain the volatile composition; rather, species identity plays a predominant role, with *D. rubrovalvata* displaying greater metabolic similarity between its tissues compared to *D. indusiata*. PCA (Fig. 4B) further resolved the sample relationships. PC1 (57.5% variance) separated cap from volva, while PC2 (22.1%) distinguished the two species within each tissue. Notably, the *D. indusiata* cap (Ccap) formed a distinct group far from *D. rubrovalvata* cap (Hcap), whereas the volva samples of both species clustered closely together near the bottom left corner of the score plot. The raw peak area values underlying these multivariate analyses are provided (Supplementary Table S5). The tight clustering of QC samples confirmed analytical reproducibility. Marginal boxplots showed that cap samples exhibited positive PC1 scores and volva samples negative scores, consistent with the distinct volatile chemistries detailed in Fig. 3. These findings provide a foundation for understanding how tissue- and species-specific biosynthetic programs contribute to the distinct aroma profiles of *Dictyophora* species.

**Fig. 4:**
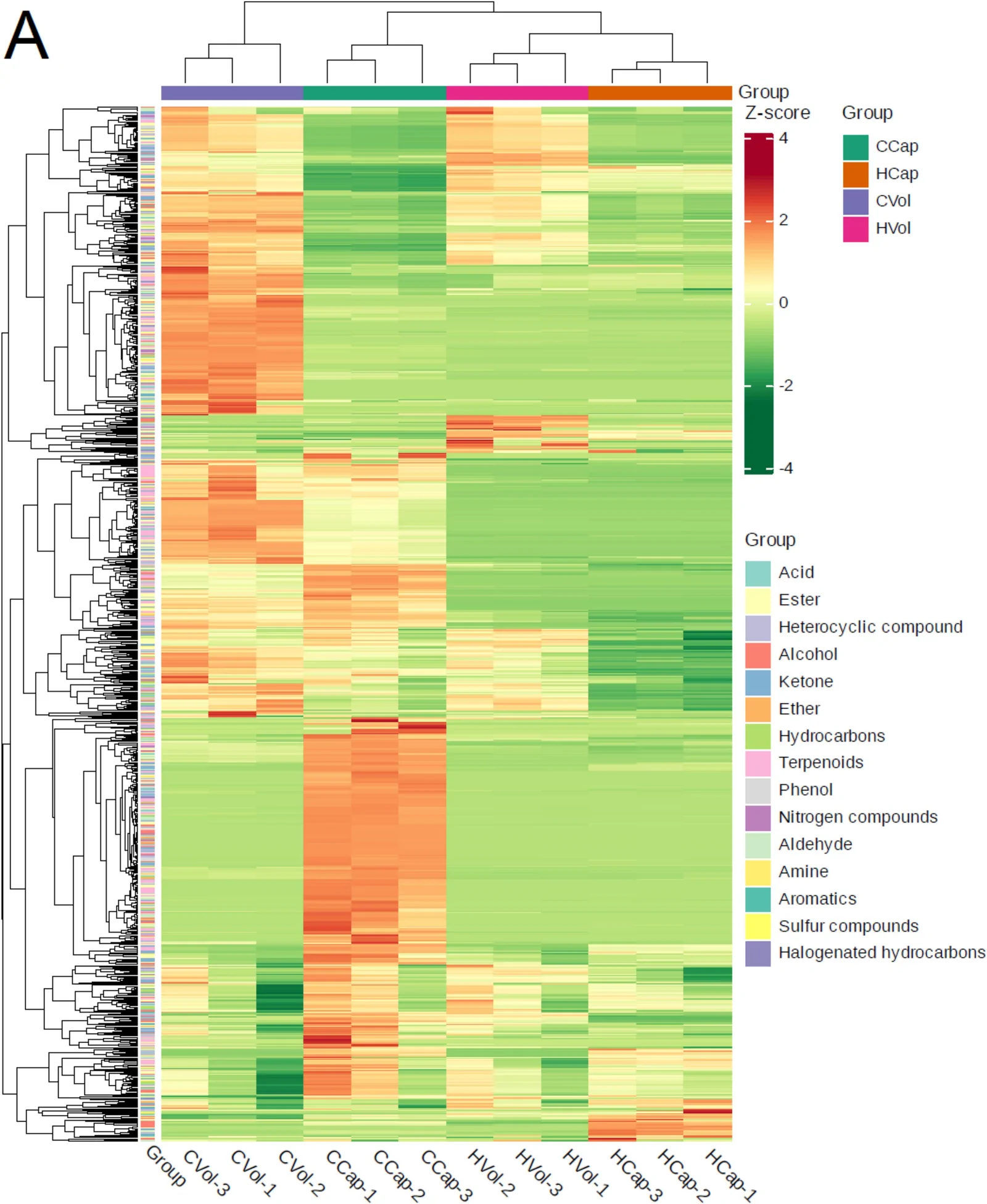

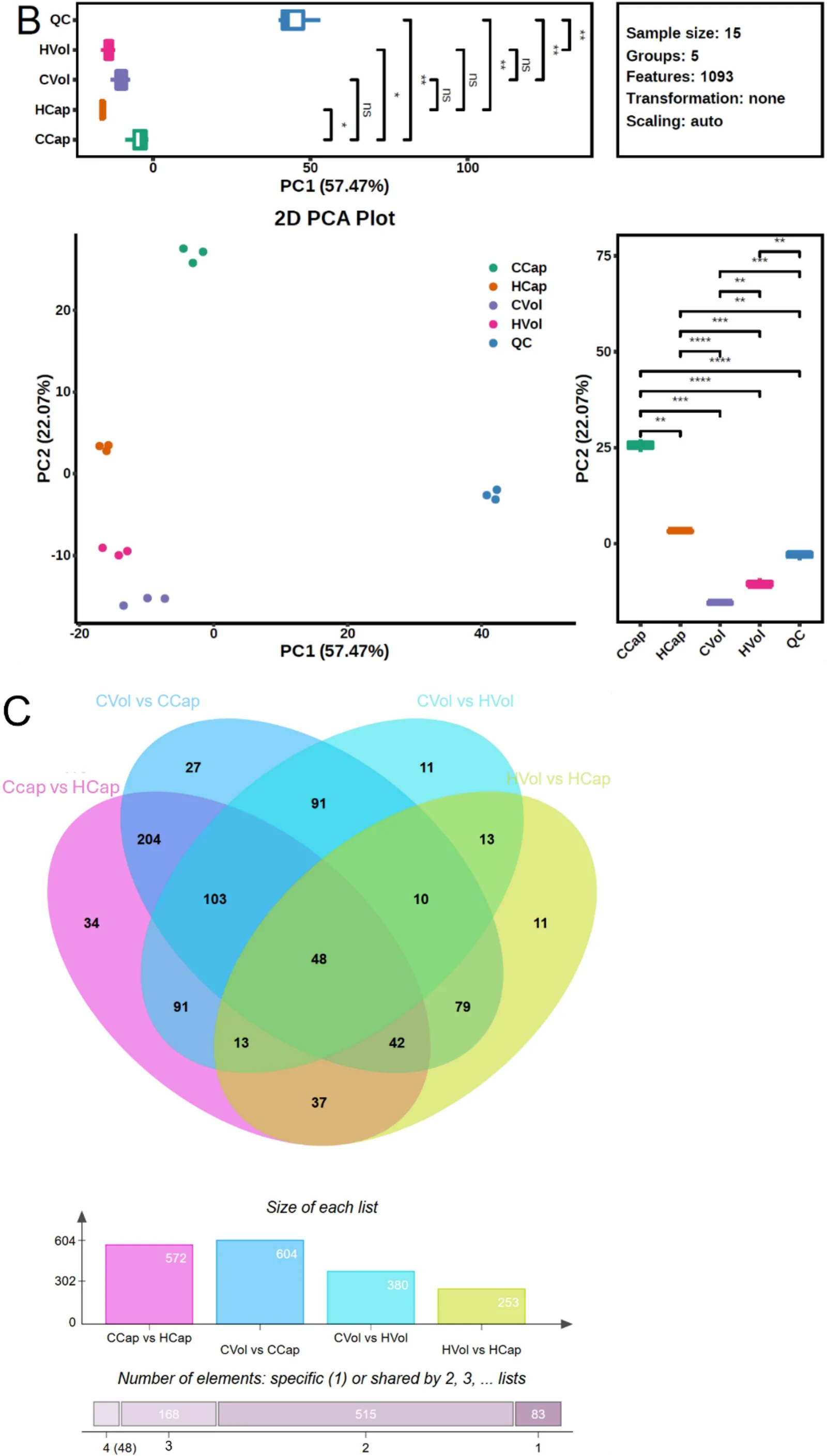

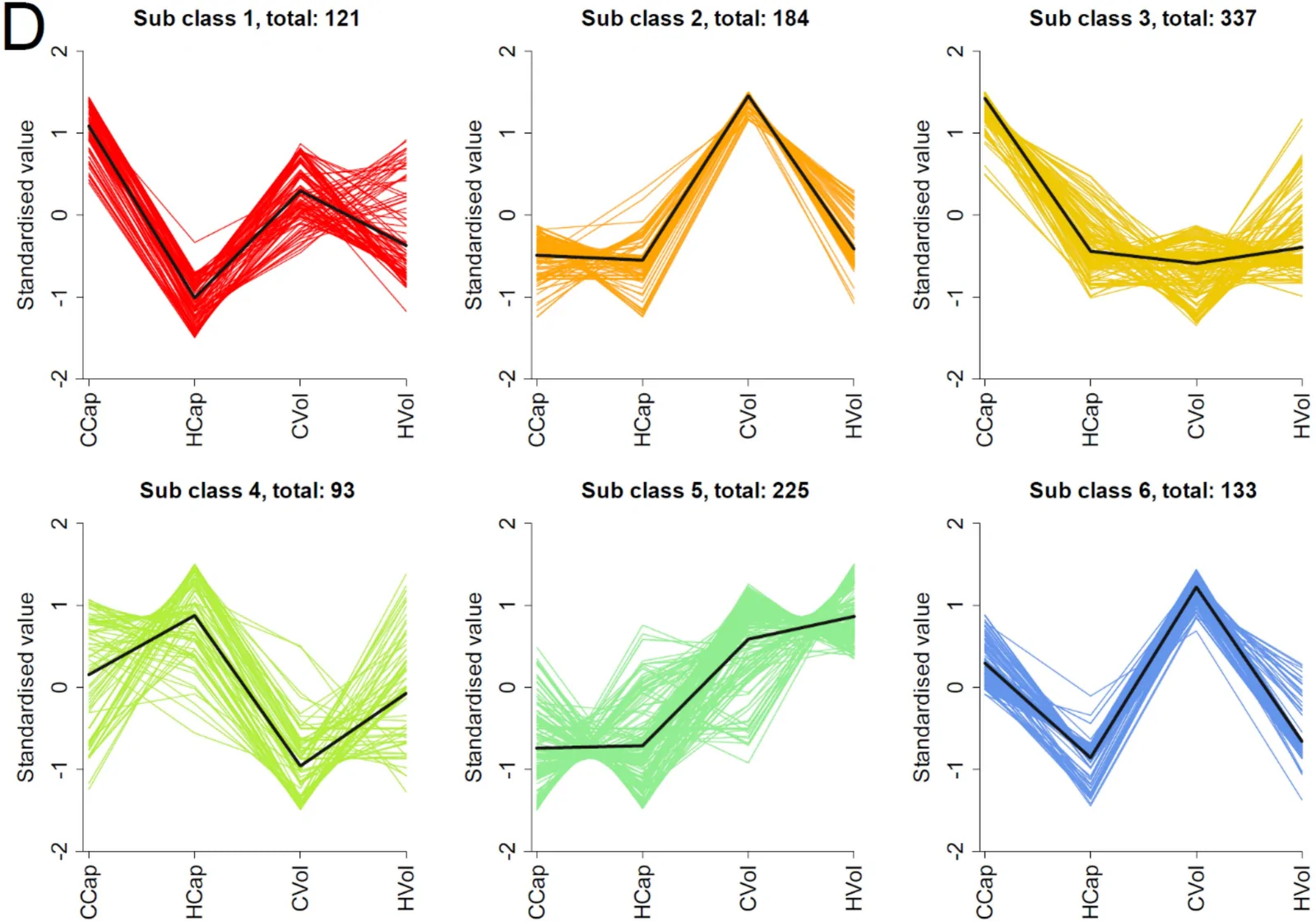
Multivariate analysis and comparative volatile profiles of cap and volva (vol) tissues from *D. indusiata* (C) and *D. rubrovalvata* (H). (A) Hierarchical clustering analysis (HCA) heatmap of all detected volatile compounds across different tissues. Color scale indicates normalized abundance. (B) Principal component analysis (PCA) score plot. (C) Venn diagrams highlights compounds in different pairwise comparisons: cap between species (Ccap vs. Hcap), volva between species (Cvol vs. Hvol), within *D. indusiata* (Ccap vs. Cvol), and within *D. rubrovalvata* (Hcap vs. Hvol). (D) K-means clustering of volatile compounds across the four samples, showing six distinct expression patterns (subclasses 1–6; Supplementary Table S6). Lines represent mean standardized abundance of compounds within each subclass across the four sample groups.

Venn analysis (Fig. 4C) quantified the overlap and uniqueness of volatile compounds across comparisons. The cap-to-cap comparison (Ccap vs. Hcap) yielded 572 differentially accumulated compounds, of which 497 were up-regulated in *D. indusiata*, highlighting substantial species divergence in the cap. The volva-to-volva comparison (Cvol vs. Hvol) showed fewer differences (380 compounds), suggesting greater metabolic conservation in the volva tissue. The smallest number of differential compounds was observed between Hvol and Hcap (253 compounds). Within *D. indusiata*, the cap vs. volva comparison (Cvol vs. Ccap) revealed the highest number of differential metabolites (604 compounds), suggesting pronounced tissue specialization in this species. Only 48 compounds were common to all four sample groups, with the Ccap vs. Hcap comparison containing the highest number of unique compounds (34), followed by Cvol vs. Ccap (27). To further evaluate the accumulation patterns of volatile compounds across tissues and species, k-means clustering was performed. The resulting clusters separated compounds into six distinct sub-classes (Fig. 4D), showing tissue- or species-specific enrichment (Supplementary Table S6). Collectively, these multivariate analyses suggest that volatile composition is shaped by both tissue identity and species background, with species exerting a stronger influence than tissue type in *D. rubrovalvata*. The greater species-level divergence observed in the cap compared to the volva suggests that the cap has undergone more specialized metabolic evolution, likely associated with ecological or sensory selection pressures.

### 3.5 OPLS-DA modeling and correlation analysis of volatile profiles

To further evaluate the discriminatory power of volatile metabolites across tissues and species, orthogonal partial least-squares discriminant analysis (OPLS-DA) was performed. Permutation testing (200 permutations, Fig. 5A) confirmed model robustness, with R^2^Y = 0.993 and Q^2^ = 0.987 (*p* < 0.005). The score plot (Fig. 5B) showed clear separation along t[1] (35.9%) and to[2] (19.9%), with *D. indusiata* cap (Ccap) and *D. rubrovalvata* cap (Hcap) distinctly separated on opposite sides. The S-plot (Fig. 5C) identified potential discriminant metabolites at the extreme ends (high |p[1]| and |p(corr)[1]|). Correlation analysis (Fig. 5D) revealed strong intra-group reproducibility (*r* > 0.95). Ccap and Hcap showed low correlation (r ≈ 0.30), indicating pronounced species-specific divergence in cap volatiles, whereas volva samples across species exhibited high correlation (r ≈ 0.95–0.98), suggesting greater metabolic conservation in the volva tissue. Based on the multivariate results, the most pronounced metabolic divergence occurs between the caps of the two species (Ccap vs. Hcap), whereas the volva tissues show greater conservation. Therefore, further detailed analysis will focus on this cap-to-cap comparison to identify species-specific discriminant volatile markers that underpin the distinct aroma profiles.

**Fig. 5:**
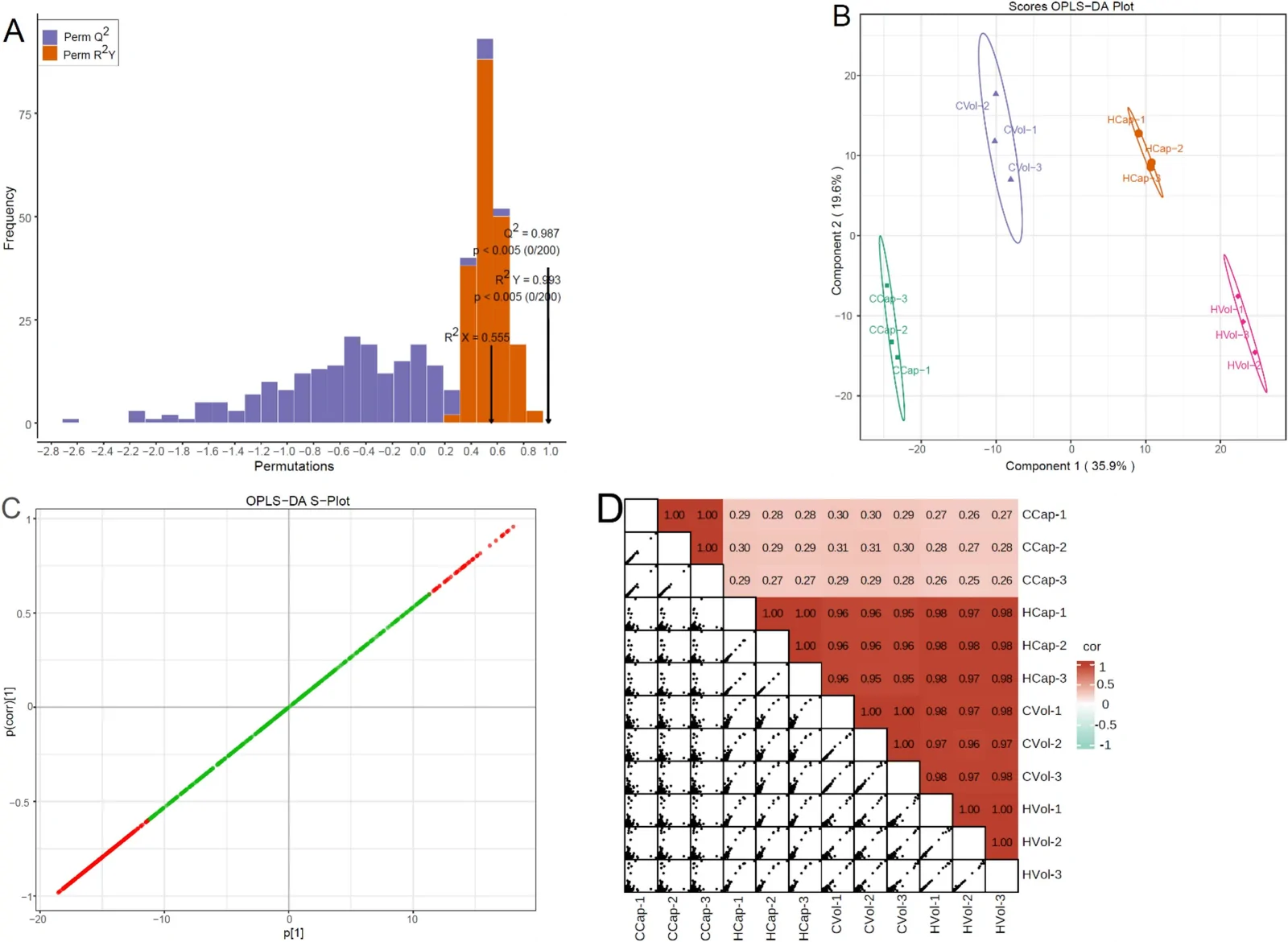
OPLS-DA modeling and correlation analysis of volatile profiles across cap and volva tissues of *D. indusiata* (C) and *D. rubrovalvata* (H). **(A)** Permutation test of the OPLS-DA model (200 permutations) showing Q^2^ (blue) and R^2^Y (orange) values. **(B)** OPLS-DA score plot, with ellipses indicating 95% confidence intervals. **(C)** S-plot displaying variable importance: metabolites (red) are potential discriminant markers (VIP > threshold). **(D)** Correlation heatmap of biological replicates, with color scale representing Pearson's correlation coefficients (r). (For interpretation of the references to color in this figure legend, the reader is referred to the web version of this article.)

### 3.6 Differential volatile compounds between caps of *D. Indusiata* and *D. Rubrovalvata*

To identify species-specific discriminant volatile markers, a detailed comparative analysis was performed between the caps of *D. indusiata* (Ccap) and *D. rubrovalvata* (Hcap). The complete list of differentially accumulated compounds with their VIP scores, fold changes, and significance values is provided (Supplementary Table S7). A total of 571 volatile compounds were significantly altered (|log₂FC| ≥ 1.0, VIP > 1.0, p<0.05), of which 461 were up-regulated in Ccap and 68 in Hcap, as illustrated in the volcano plot (Fig. 6A). The pronounced asymmetry in up-regulated compounds (461 vs. 68) indicates a substantially richer volatile repertoire in *D. indusiata* cap. The class-level scatter plot (Fig. 6B) further resolved the chemical families associated with this divergence. Terpenoids, esters, alcohols, and heterocyclic compounds were among the most enriched classes in Ccap, with many compounds exhibiting log₂FC values exceeding 5.0. In contrast, Hcap showed enrichment in fewer classes, with modest fold changes.

**Fig. 6:**
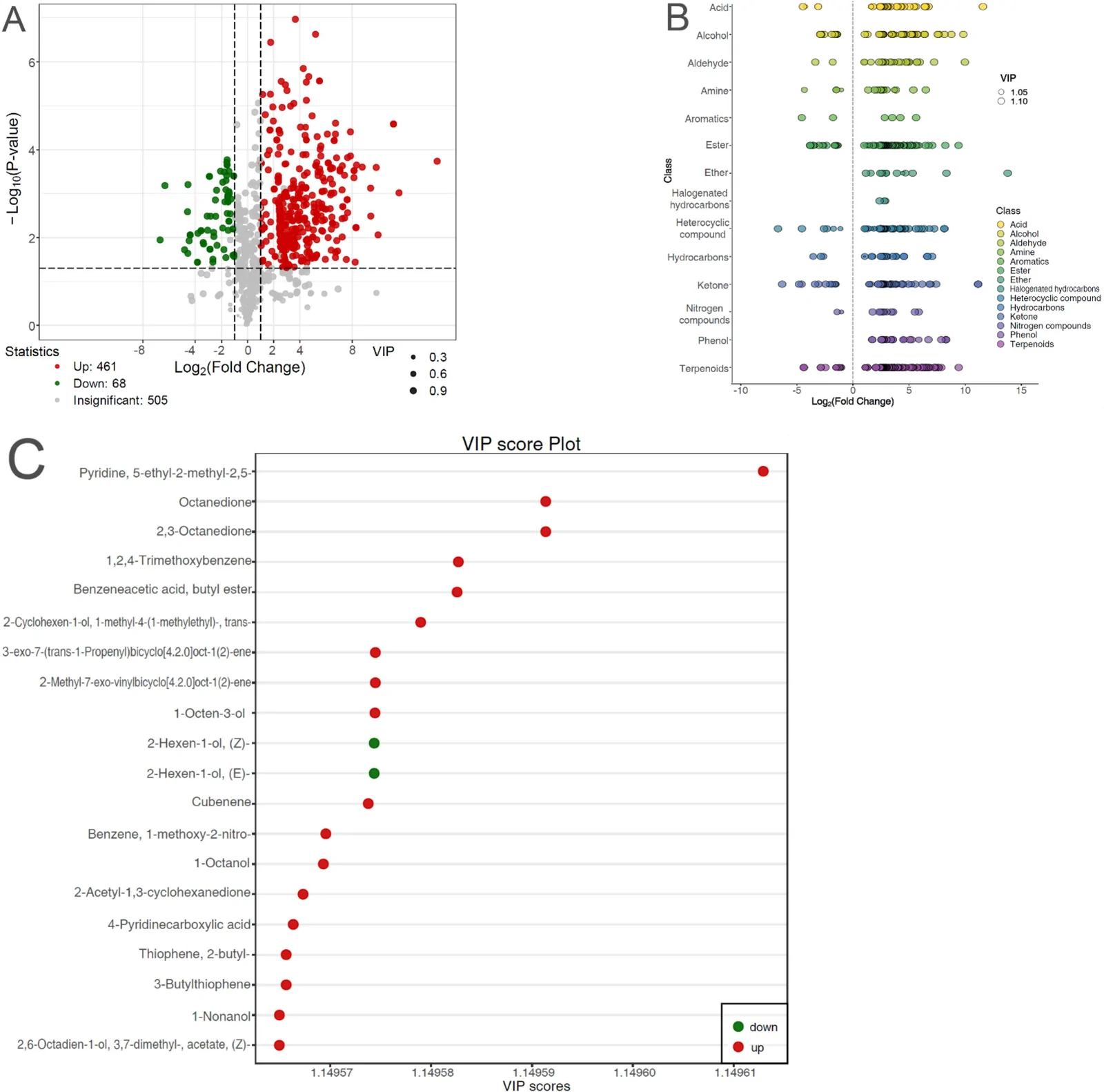

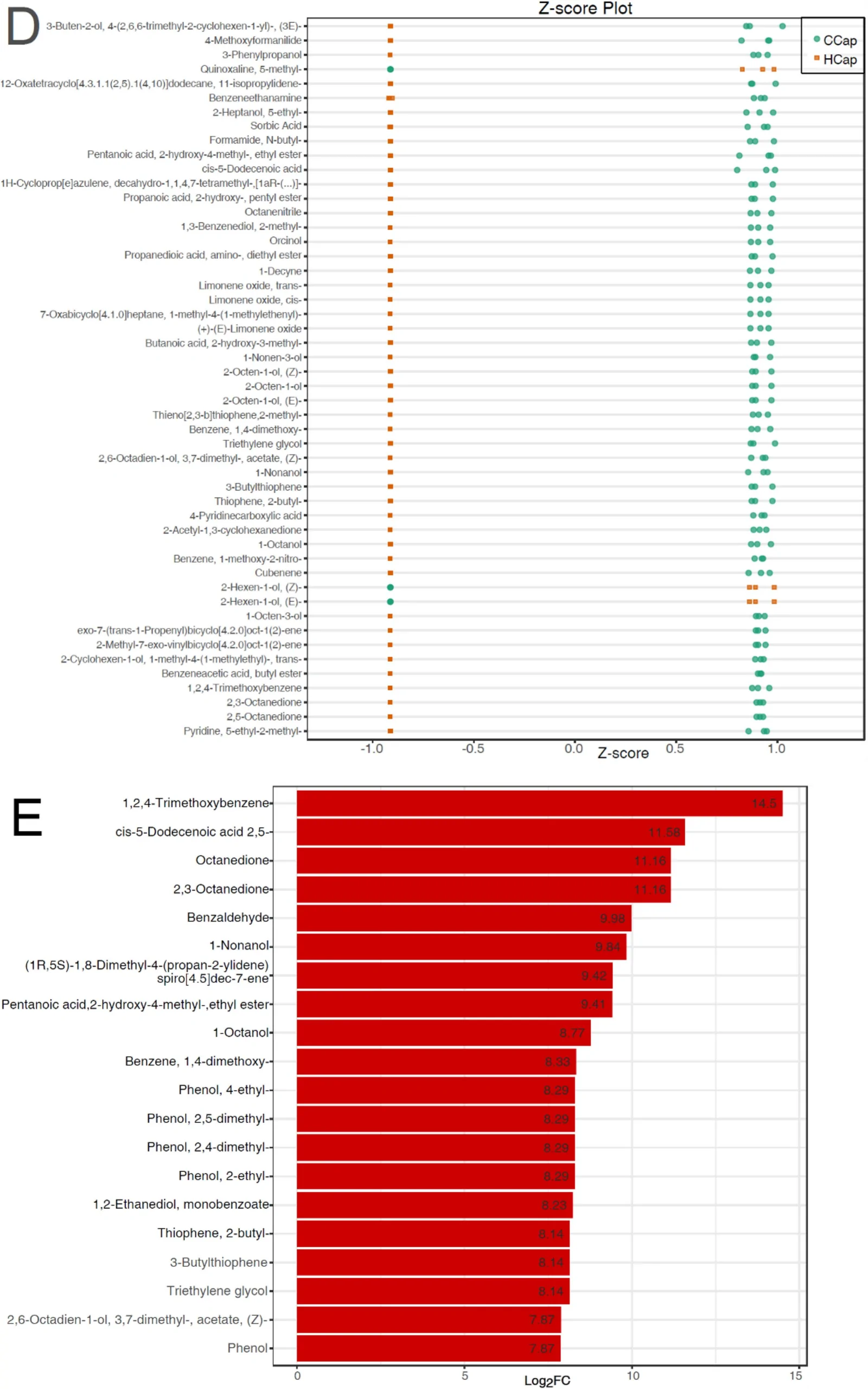
Differential volatile compounds between *D. indusiata* cap (Ccap) and *D. rubrovalvata* cap (Hcap). (A) Volcano plot showing log₂ fold change on axis and log₁₀(p-value) on y-axis. Compounds with VIP > 1 are highlighted in color (red: up-regulated in Ccap; green: up-regulated in Hcap). (B) Class-level heatmap illustrating the relative enrichment of major chemical families showing log₂ fold change. (C) Score-plot of the top 20 compounds ranked by variable importance in projection (VIP) score. (D) *Z*-score heatmap of the top 50 VIP compounds, showing normalized abundance across biological replicates. (E) Bar chart of the top 20 compounds ranked by log₂ fold change; values are annotated on each bar. (For interpretation of the references to color in this figure legend, the reader is referred to the web version of this article.)

The VIP bar chart (Fig. 6C) ranked the top 20 discriminant compounds by VIP score, with terpenoids and esters dominating the list. The *Z*-score heatmap (Fig. 6D) displayed the normalized abundance of the top 50 VIP compounds across replicates, confirming consistent up-regulation of these markers in Ccap relative to Hcap. Finally, the top 20 log₂FC bar chart (Fig. 6E) highlighted the most dramatically altered compounds, with several terpenoids and esters showing fold changes exceeding 10-fold. Collectively, these analyses suggest that *D. indusiata* cap possesses a more diverse and quantitatively richer volatile profile than *D. rubrovalvata* cap, associated with elevated terpenoids and esters. These compounds may represent promising species-specific discriminant markers associated with the distinct aroma characteristics of the two species.

### 3.7 Flavor-active compound profiling between caps

To translate chemical differences into sensory-relevant attributes, flavor-active compounds were analyzed based on odor descriptions and relative abundances. The radar chart showed that *D. indusiata* cap (Ccap) was predicted to have higher compound counts for sweet, fruity, woody, floral, and herbal notes, while *D. rubrovalvata* cap (Hcap) showed higher green and fatty attributes (Fig. 7A). Sweet (61 compounds), green (60), and fruity (57) were the most abundant predicted descriptor categories (Supplementary Table S8).

**Fig. 7:**
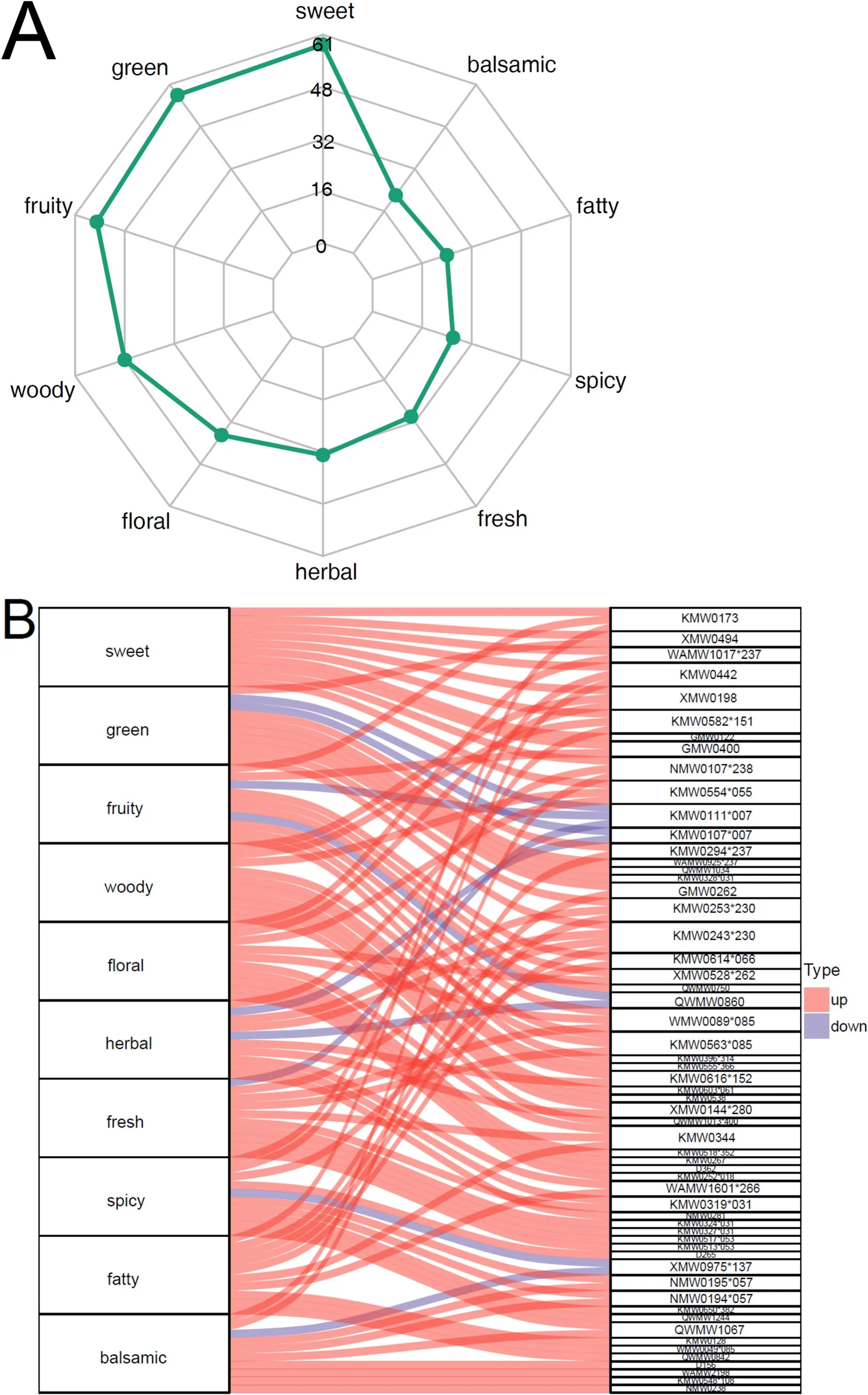

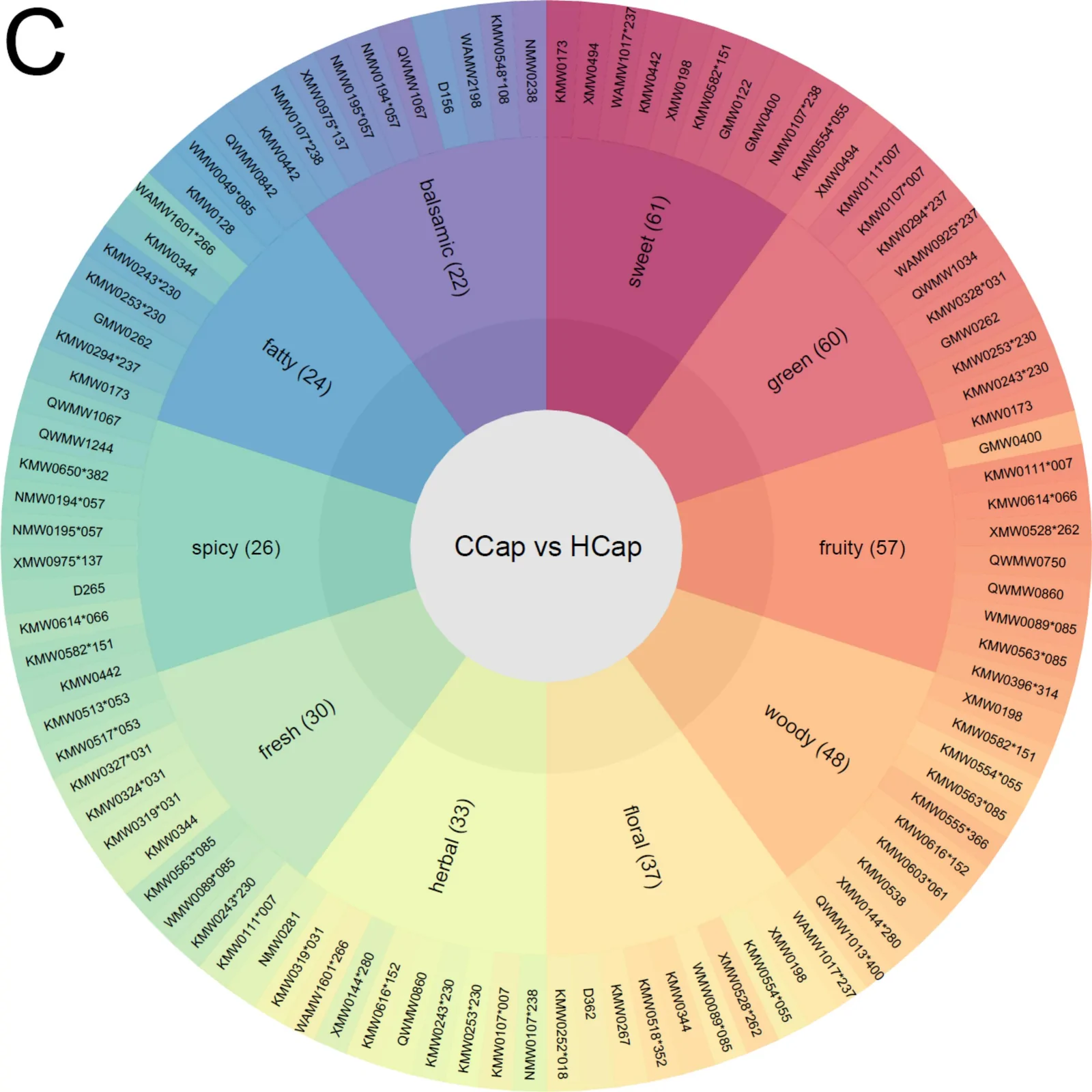
Flavor-active compound profiling between *D. indusiata* cap (CCap) and *D. rubrovalvata* cap (HCap). (A) Radar chart showing the number of volatile compounds associated with each sensory descriptor (e.g., sweet, fruity, green). Numerical scales indicate the number of compounds. (B) Sankey diagram illustrating the flow of upregulated (red) and downregulated (blue) volatile compounds to their associated sensory descriptors (e.g., sweet, fruity, green), with stream width proportional to the number of compounds. (C) Sunburst chart depicting the hierarchical organization of flavor compounds, where sector size reflects the number of compounds associated with each sensory descriptor (e.g., sweet = 61, green = 60). The outer ring lists the individual compound identifiers. (For interpretation of the references to color in this figure legend, the reader is referred to the web version of this article.)

The Sankey diagram illustrated chemical class contributions to flavor compounds (Fig. 7B). Terpenoids and esters were predominant associated with sweet, fruity, and floral notes, with compounds such as 1-octen-3-ol (KMW0173), α-ionone (KMW0554*055), 2-octen-1-ol, (*Z*)- (WAMW1017*237), and various limonene oxides enriched in Ccap. Notably, (*E*)-2-hexen-1-ol and (Z)-2-hexen-1-ol, associated with green and herbal aromas, were higher in Hcap. The sunburst chart showed sweet (61) and green (60) as the most represented descriptors in the inner ring (Fig. 7C). The outer ring listed discriminant compounds, including 1-octen-3-ol (KMW0173) (mushroom, earthy) associated with green, fresh, and fatty notes, and terpenoids uniquely enriched in Ccap: α-ionone (KMW0554*055) (floral, woody), 2 undecenal (KMW0513*053) (fresh), E 2 undecenal (KMW0517*053) (fresh), and cis−/trans-limonene oxide (fresh). Collectively, *D. indusiata* cap is predicted to possess a more complex aroma profile dominated by sweet, fruity, and floral notes associated with terpenoids and esters, whereas *D. rubrovalvata* cap is predicted to exhibit simpler characteristics with pronounced green and fatty nuances.

### 3.8 Correlation and network analysis of discriminant volatile compounds

The chord diagram (CCap vs HCap) displayed the top 50 discriminant compounds (VIP > 1) arranged along the outer ring (Supplementary Table S9), with point size representing absolute log₂FC and color indicating chemical class (Fig. 8A). Most compounds, including terpenoids (e.g., α-ionone, β-guaiene, limonene oxides), esters (e.g., allyl nonanoate, 2-octenoic acid ethyl ester), and alcohols (e.g., 1-octen-3-ol), formed a large, densely connected cluster with strong positive correlations (red chords), consistent with their coordinated enrichment in *D. indusiata* cap. In contrast, three compounds such as quinoxaline, 5-methyl (GMW0301), (E)-2-hexen-1-ol, and (Z)-2-hexen-1-ol, showed predominantly negative correlations (blue chords) with the main cluster, consistent with their higher abundance in *D. rubrovalvata* cap. The flavor network revealed distinct associations between discriminant volatile compounds and flavor descriptors (Fig. 8B; Supplementary Table S8). Terpenoids and esters (e.g., α-ionone, β-guaiene, limonene oxides) were predominantly associated with sweet, fruity, and floral notes, consistent with their enrichment in *D. indusiata* cap. Conversely, green-note alcohols such as (E)- and (Z)-2-hexen-1-ol were prominently associated with green and herbal descriptors, aligning with their higher abundance in *D. rubrovalvata* cap.

**Fig. 8:**
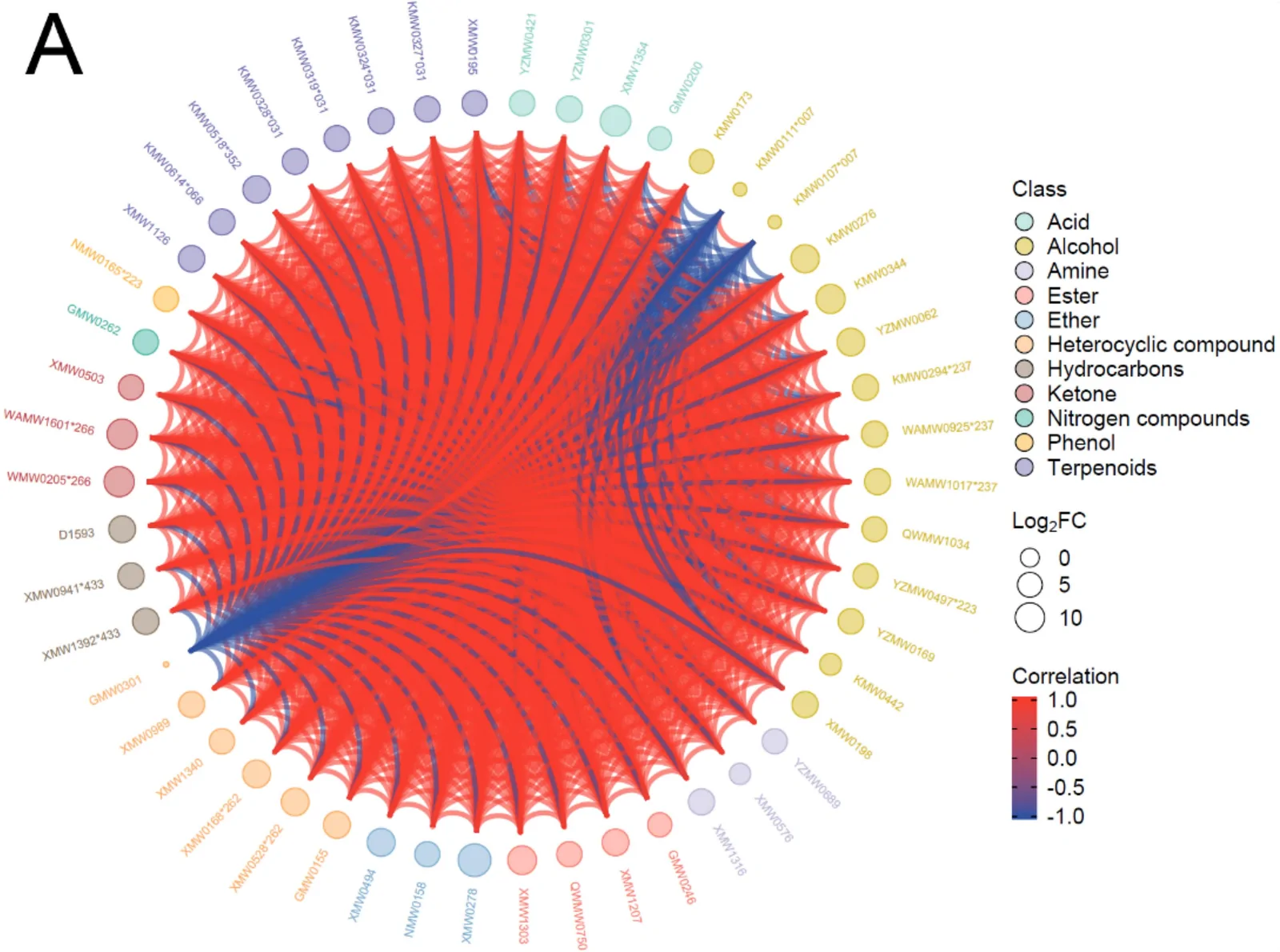

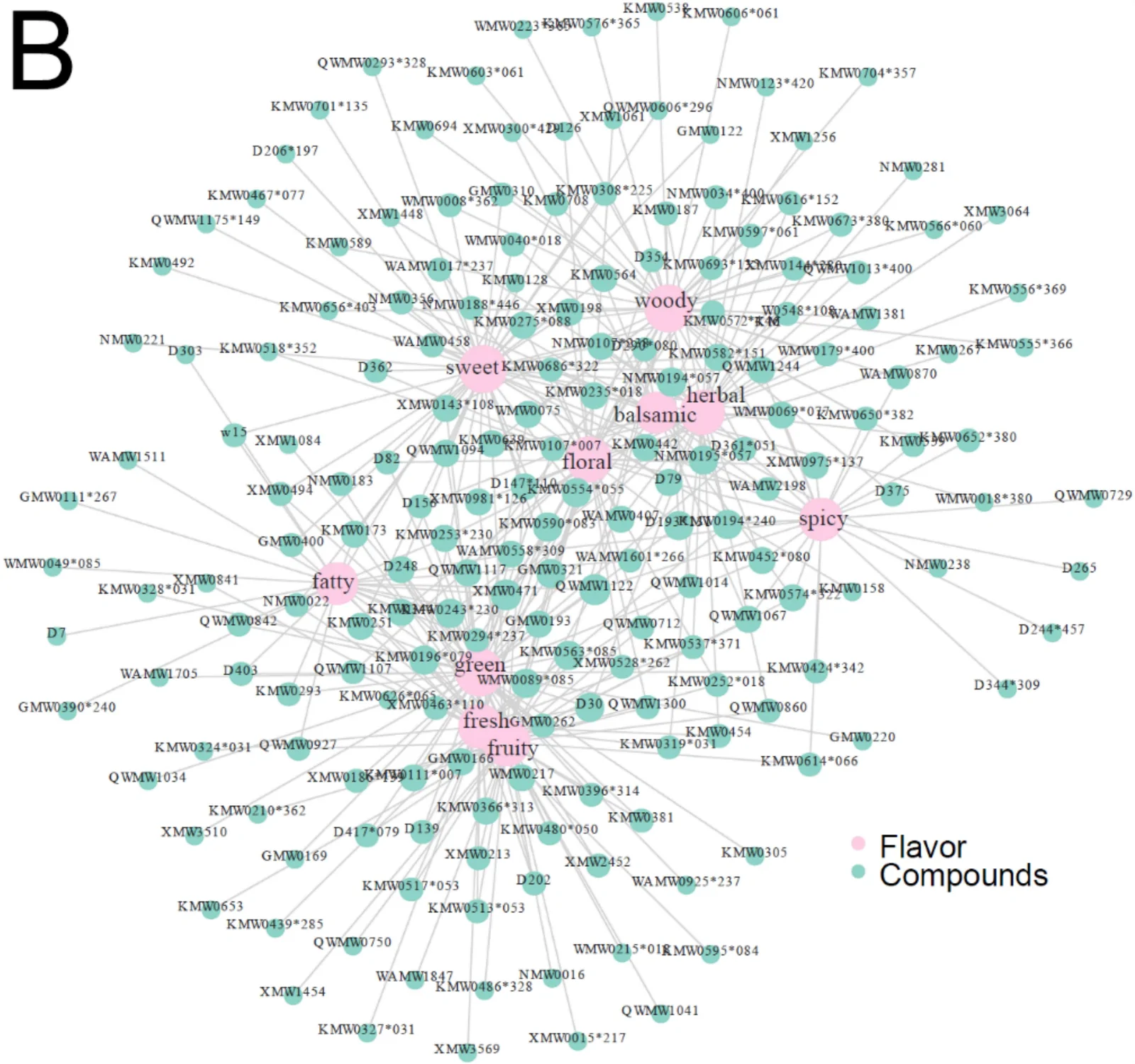

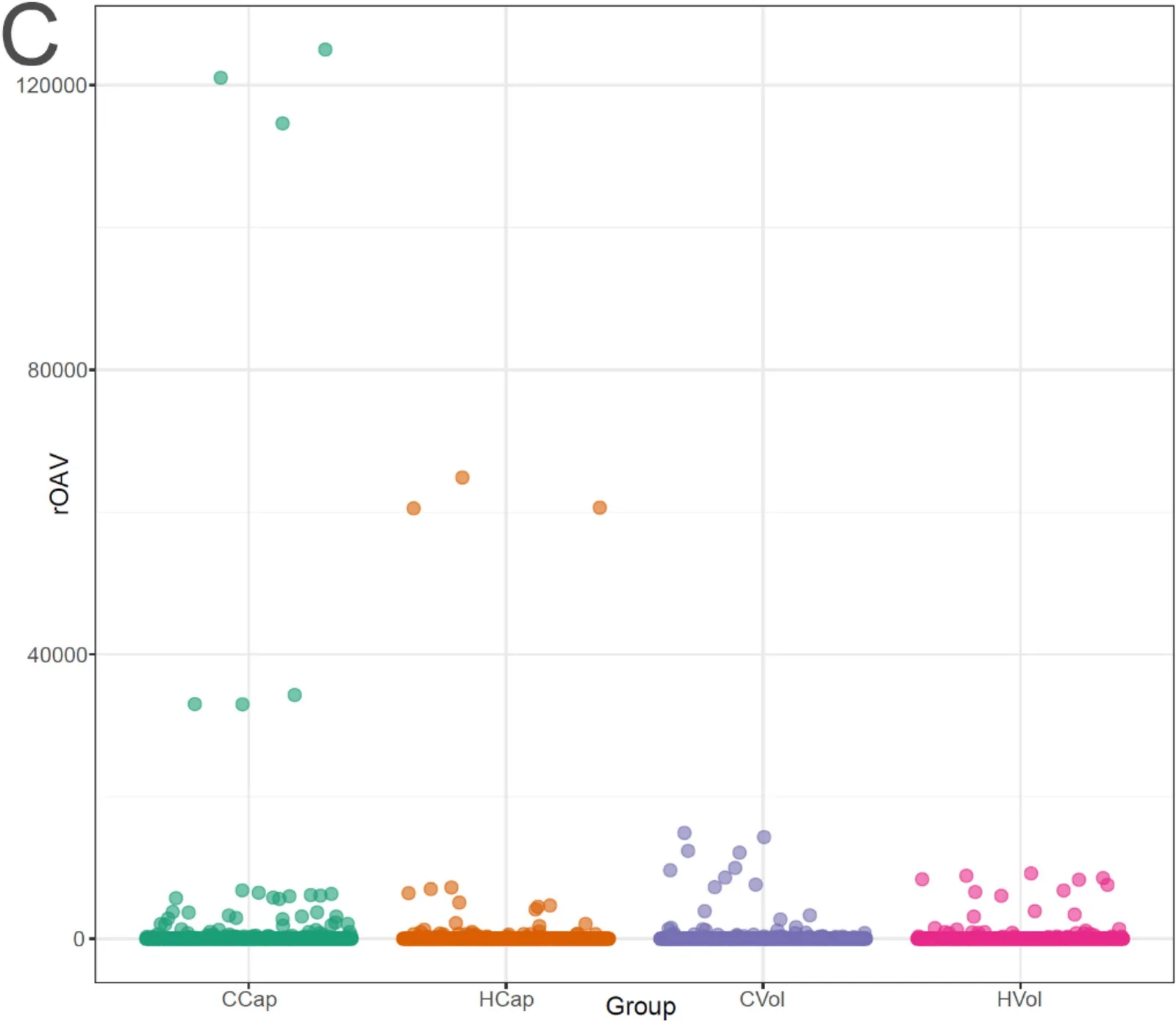
Correlation and network analysis of volatile compounds. (A) Chord diagram (CCap vs HCap) displaying the top 50 compounds ranked by VIP, point size represents log₂FC magnitude; colors indicate chemical classes. Red chords denote positive correlations (Pearson's r>0.8), blue chords denote negative correlations (r<−0.8). (B) Correlation network of volatile compounds, where nodes represent compounds (color-coded by chemical class) and edges indicate significant Pearson correlations (∣r∣>0.8, p<0.05). (C) Relative odor activity values (rOAV) of key aroma compounds across the four sample groups, with values scaled to the maximum within each compound. (For interpretation of the references to color in this figure legend, the reader is referred to the web version of this article.)

The rOAV scatter plot displayed the distribution of aroma active compounds (rOAV ≥1) across the four sample groups (Fig. 8C). Ccap exhibited the highest number of compounds with elevated rOAV values, particularly terpenoids (e.g., α-ionone, limonene oxide) and esters (e.g., ethyl (E)-2-octenoate). 1-Octen-3-ol (mushroom, earthy) showed exceptionally high relative abundance in Ccap, suggesting its dominant sensory contribution. Hcap displayed moderate rOAV values with notable contributions from green note alcohols such as (E)- and (Z)-2-hexen-1-ol, consistent with its predicted greener aroma profile. Volva samples exhibited overall lower rOAV values, with Cvol showing moderate levels for certain terpenoids (e.g., α-ionone) and Hvol the lowest overall. Collectively, these analyses suggest that discriminant volatile compounds form distinct flavor-associated networks, with terpenoids and esters associated with sweet and fruity notes in *D. indusiata* cap, while green note alcohols contribute to the greener profile of *D. rubrovalvata* cap. The rOAV data further suggests that *D. indusiata* cap has the highest aroma potency, indicating its potential superior flavor quality.

### 3.9 Phenolic metabolite profiling

To elucidate the observed differences in total phenolic content and radical scavenging capacity (Fig. 1B-E), UPLC-MS metabolomics was performed. Among the differential phenolic compounds (Ccap vs. Hcap), the majority were upregulated in *D. indusiata* cap (Supplementary Table S10; Fig. 9A). At MSI Level 1, key upregulated compounds included alpha-oxo-benzeneacetic acid (log₂FC = 13.48, VIP > 1.125), veratraldehyde (log₂FC = 10.74, VIP = 1.125), phenylpropanoid derivatives such as phenyllactate (log₂FC = 9.16), and phloretate (log₂FC = 7.97) and DL-3-phenyllactic acid (log₂FC = 7.88, VIP = 1.12). All other phenolic compounds were identified at MSI Level 2 or 3 (putative or tentative identification) and are listed in Supplementary Table S10. Similarly, in the volva (Cvol vs. Hvol), MSI Level 1 compounds enriched in *D. indusiata* volva included alpha-oxo-benzeneacetic acid (log₂FC = 8.79), phenyllactate (log₂FC = 7.58), veratraldehyde (log₂FC = 7.37, VIP = 1.12), phloretate (log₂FC = 7.32, VIP = 1.12), and DL-3-phenyllactic acid (log₂FC = 7.27, VIP = 1.12) (Supplementary Table S11; Fig. 9B). Downregulated (in Cvol) compounds included 3,4-dihydroxybenzoate (log₂FC = −1.75), 2,5-dihydroxybenzoate (log₂FC = −1.94), trans-2-hydroxycinnamate (log₂FC = −3.38), and trans-3-hydroxycinnamate (log₂FC = −3.49) (Supplementary Table S11). Moreover, across four comparisons (Fig. 9C), Ccap vs. Hcap yielded the highest number of differential phenolics (109), with 81 up-regulated in *D. indusiata* cap. Cvol vs. Hvol showed 84 differential compounds, with 52 up-regulated in *D. indusiata* volva. Within *D. indusiata*, Cvol vs. Ccap showed 91 differential phenolics (55 down-regulated in volva), reflecting tissue-specific accumulation patterns.

**Fig. 9:**
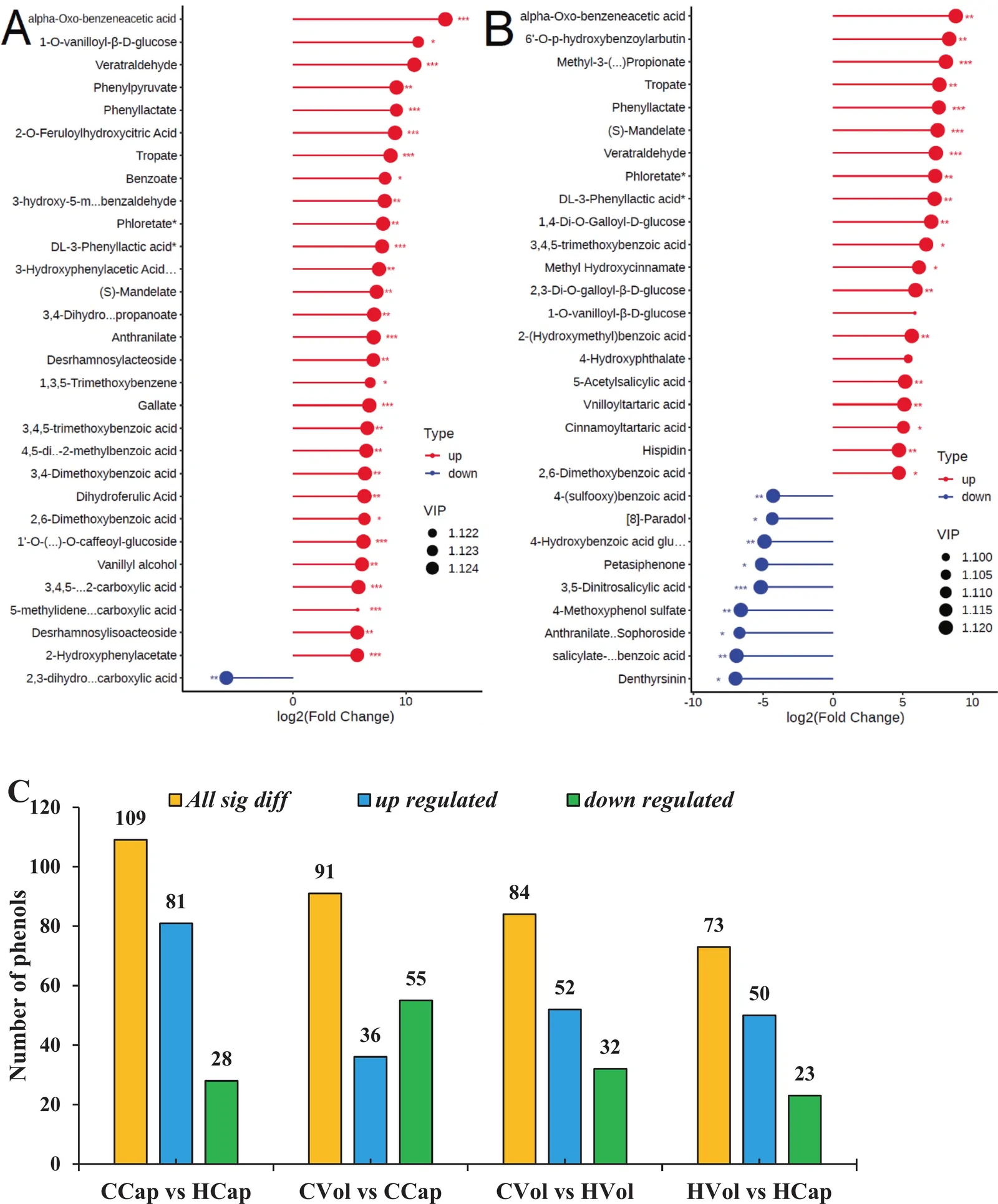
Phenolic metabolite profiling in cap and volva tissues of *D. indusiata* (C) and *D. rubrovalvata* (H). (A) Lollipop plot showing the top 30 differential phenolic compounds in Ccap vs. Hcap. Circle size represents variable importance in projection (VIP) score; bar length represents log₂ fold change. Compounds with VIP > 1 and p<0.05 are shown. (B) Lollipop plot showing the top 30 differential phenolic compounds in Cvol vs. Hvol. (C) Column graph summarizing the number of differentially abundant phenolic compounds across four comparisons.

These phenolic profiles suggest that *D. indusiata* possesses a richer phenolic repertoire in both cap and volva compared to *D. rubrovalvata*, with the cap showing the most pronounced enrichment. This evidence is consistent with the higher total phenolic content and radical scavenging capacity measured biochemically (Section 3.1), indicating species-specific metabolic specialization in phenolic biosynthesis. Collectively, the identified phenolic and volatile compounds provide a chemical basis for the radical scavenging capacity and predicted flavor qualities of *D. indusiata* cap, suggesting its potential as a high-value functional food ingredient.

## 4 Discussion

### 4.1 Higher radical scavenging chemotype of *D. Indusiata* cap

The present work suggests that the cap of *D. indusiata* represents a distinct radical scavenging chemotype among the tissues and species examined. Its higher radical scavenging capacity (DPPH• and ABTS•+) aligns with UPLC-MS evidence of enriched phenolic acids and phenylpropanoids (e.g., alpha-oxo-benzeneacetic acid, phenyllactate, phloretate, and vanillate) (Fig. 1, Fig. 9). These structures contain multiple ortho-dihydroxyl or trihydroxyl groups and extended conjugation, which lower redox potential and enhance electron donation and metal chelation compared to monophenolics a structure–activity relationship well documented for polyphenolic compounds in tea, cereals, and fruits (Chen et al., 2024; Duan et al., 2025; Xu & Wang, 2025). Unlike common cultivated mushrooms (*Agaricus bisporus*, *Pleurotus ostreatus*), which typically harbor low levels (<1–2 mg/g) of simple phenolic acids (Abdelshafy et al., 2022; Elhusseiny et al., 2021), *D. indusiata* cap displays a richer and more diversified phenolic repertoire, including confirmed phenolic acids and phenylpropanoids (Supplementary Table S10). Based on known mechanisms, these phenolics can scavenge reactive species and, in other systems, activate Nrf2-dependent defenses (Rao & Zheng, 2025; Rudrapal et al., 2024). We utilized DPPH and ABTS^+^ assays, which primarily measure single-electron transfer capacity. We acknowledge that hydrogen atom transfer mechanisms, as measured by oxygen radical absorbance capacity (ORAC) and cellular-level antioxidant activity (CAA) are not captured here. Given that assay method significantly influences measured activity (Adebayo et al., 2018; Smolskaitė et al., 2015), future studies should incorporate ORAC and CAA assays to provide a complete functional profile of *Dictyophora* phenolics.

Similar radical scavenging functions have been linked to specific transcription factor modules in plants under oxidative stress (Zhang et al., 2025). Although the in-vitro radical scavenging capacity of *D. indusiata* cap phenolics is well demonstrated, we acknowledge that the bioavailability of complex phenolic conjugates remains unknown. Simulated gastrointestinal digestion studies are required to assess whether these compounds survive the digestive process and reach target tissues in bioactive form. Recently, Wang et al. (2024) developed an in-vitro dynamic digestion model for *D. indusiata* polysaccharides, demonstrating that simulated salivary-gastrointestinal digestion alters the structural characteristics of these macromolecules (Wang, Zeng, et al., 2024). Similar approaches applied to phenolic fractions would be necessary to evaluate the in-vivo potential of the compounds identified here. Nevertheless, the in-vitro radical scavenging capacity provides a foundation for prioritizing *D. indusiata* cap for further bioavailability assessment (K. Kumar et al., 2021; Yang et al., 2025). To ensure the highest confidence in the reported biomarkers, only compounds identified at MSI Level 1 are discussed in the main text; all compounds detected are available in the Supplementary Tables (S2, S10, S11).

### 4.2 Tissue-specific carbon partitioning and taste-active metabolites

Marked tissue-specific differences in primary metabolism were observed between the two species, with direct consequences for taste. *D. rubrovalvata* cap accumulated substantially higher levels of sugars and glutamic acid than *D. indusiata* cap, whereas *D. indusiata* volva was enriched in glucose, soluble sugar, and especially malic acid (Fig. 2). This pattern suggests divergent carbon partitioning: *D. rubrovalvata* channels carbon into readily available carbohydrates and free amino acids in the cap, while *D. indusiata* stores a larger fraction as organic acids and starch in the volva tissue, a strategy also observed in engineered plants for stress resilience (Zhao et al., 2025).

Spatial heterogeneity in carbohydrate and organic acid pools is common in Basidiomycetes, reflecting localized demands for growth, turgor maintenance, and spore dispersal (Bhattacharjee et al., 2023; Ye et al., 2026). The high sugar and glutamate content in *D. rubrovalvata* cap may contribute to enhanced sweetness and umami perception, with glutamate synergizing with 5′-ribonucleotides for savory notes (Xia et al., 2025). This tissue-specific enrichment of glutamic acid in *D. rubrovalvata* cap is consistent with previous findings. Liang et al. (2020) reported that glutamic acid was the most abundant free amino acid in *D. rubrovolvata* fruit bodies (1.34–4.86 mg/g), and Wang, Han, et al. (2024) confirmed that glutamic acid dominated the free amino acid pool across embryo, colloid, and fruiting body tissues. Our cap-specific value (4.56 mg/g DW) falls within the upper range of these earlier whole-fruiting-body reports, suggesting that the cap is a site of preferential glutamic acid accumulation. In contrast, the lower glutamic acid content in *D. indusiata* cap (2.13 mg/g DW) is closer to the 0.54 mg/g previously reported for whole *D. indusiata* fruiting bodies (Mau et al., 2001). This species-level contrast in glutamic acid allocation further supports the proposed chemotype distinction: *D. rubrovalvata* cap is optimized for umami-driven flavor, whereas *D. indusiata* cap prioritizes phenolic and volatile secondary metabolism.

We note that volatile compounds were quantified on a fresh weight basis while primary metabolites and phenolics were on a dry weight basis. This reflects the different analytical workflows (fresh headspace SPME vs. lyophilized extraction) and is standard in flavoromics. For dried-product applications (e.g., soups, seasonings), volatile content should be recalculated using appropriate moisture conversion factors (Meng et al., 2023; Zheng et al., 2024).

### 4.3 Volatile divergence and aroma-active markers

The volatile metabolome revealed a clear dichotomy: *D. indusiata* cap drives aroma complexity, with total volatile content roughly double that of *D. rubrovalvata* cap, dominated by terpenoids, esters, alcohols, ethers, and heterocyclics (Fig. 7, Fig. 8). rOAV analysis suggested that sensory impact comes from a small subset of compounds. In *D. indusiata* cap, 1-octen-3-ol, the classic “mushroom” odorant, showed exceptionally high relative abundance, while α-ionone (violet, woody, fruity) also exhibited markedly elevated levels. Together with limonene oxides and fruity esters like ethyl (*E*)-2-octenoate, these high-impact odorants may explain the sweet, fruity, floral, and woody notes suggested for *D. indusiata* cap, based on rOAV analysis. In contrast, *D. rubrovalvata* cap exhibited a simpler profile with lower terpenoids and esters, and a relative dominance of C6 green-note alcohols—(E)- and (Z)-2-hexen-1-ol. These compounds, derived from lipoxygenase-mediated oxidation of linolenic acid, are reported to impart green, grassy, and herbal nuances, aligning with the predicted greener aroma of this species (Vincenti et al., 2019).

A striking feature of *D. indusiata* cap was the high abundance of ethers, nearly absent in *D. rubrovalvata* (Fig. 3D). Ethers are rarely emphasized in mushroom aroma studies but can contribute sweet, floral, or spicy notes at low thresholds (Deng et al., 2024). Their strong species- and tissue-specific enrichment suggests they could serve as a chemotaxonomic marker within *Dictyophora* and related Phallaceae (Liu et al., 2024; Yang et al., 2024). Overall, *D. indusiata* cap suggests a complex, intense aroma blending mushroom, fruity, and floral notes, while *D. rubrovalvata* cap suggests a cleaner, greener, and fattier profile (Fig. 7B), consistent with reports from other sensory studies, although direct sensory validation was not performed here.

We acknowledge that our aroma descriptors (e.g., sweet, fruity, floral) are inferred from rOAV analysis and literature-based odor thresholds rather than from direct sensory evaluation. Trained descriptive sensory panels are required to validate these predicted aroma profiles, as matrix effects and perceptual interactions can substantially modulate actual human odor perception. A recent study by Cheng et al. (2026) employed trained panelists using descriptive analysis and consumers using check-all-that-apply (CATA) to evaluate chicken soups containing *D. indusiata*, demonstrating that this species exhibits a distinct sensory profile compared to other edible fungi (Cheng et al., 2026). Future studies integrating such sensory methodologies with the chemical data presented here will be essential for confirming the predicted aroma complexity of *D. indusiata* cap.

### 4.4 Tissue- and species-specific metabolic specialization

Multivariate analyses (HCA, PCA, OPLS-DA) showed that volatile composition is shaped by both tissue and species, but the degree of divergence differs markedly between caps and volvas (Fig. 4, Fig. 5). Caps of the two species separated strongly, whereas volvas clustered more closely, indicating that the cap is the primary site of species-specific metabolic specialization. Within *D. indusiata*, cap and volva also differed more than within *D. rubrovalvata*, suggesting stronger tissue differentiation in the former.

This pattern aligns with the ecological role of the cap in Phallaceae and other Basidiomycetes. The cap houses the gleba and attracts insect or animal dispersers via strong odors, a strategy tightly linked to volatile production (El-Gharabawy, 2023; Rungjindamai & Jones, 2024). The richer, more diverse volatile profile of *D. indusiata* cap, including high-impact terpenoids and esters (Fig. 6), may reflect adaptation to different ecological niches, but this remains to be tested. In contrast, the volva serves primarily structural and protective functions, requiring a more conserved metabolic program focused on storage and defense. This is supported by the high correlation of volva volatiles between species and fewer differential compounds. Similar tissue-biased specialization occurs in *Agaricus* and *Pleurotus*, where caps exhibit higher levels of aroma-active volatiles and phenolics than stipes, and respond more strongly to environmental cues (Deng et al., 2024; Jiang et al., 2023). Our data suggest that, in *Dictyophora*, the cap (a reproductive tissue) exhibits greater metabolic divergence than the volva, consistent with the idea that reproductive structures may be more evolutionarily labile.

### 4.5 Coordinated regulation of phenolic and volatile pathways

A notable observation from this study is the concurrent enrichment of phenolic and volatile biosynthetic products in *D. indusiata* cap. The same tissue that accumulates phenylpropanoids also shows the highest levels of terpenoids, esters, and key aroma-active alcohols. Phenylpropanoids derive from the shikimate pathway, terpenoids from mevalonate/MEP pathways, and esters from esterification reactions, pathways that share cofactors (NADPH, ATP) and may be metabolically linked (L. Chen et al., 2026; Paul et al., 2023). In mushrooms, phenolics and volatile terpenoids often increase together during stress or maturation, suggesting a possible shared metabolic program (Paul et al., 2023). Similarly, in cereals, jasmonate signalling coordinates defense-related secondary metabolism, including phenolic and volatile compounds (Xu et al., 2026). For *D. indusiata*, the cap may act as a metabolic “hotspot” where phenylpropanoids contribute to free radical scavenging protection while terpenoids and esters serve olfactory functions. The positive correlations among discriminant volatiles in network analyses support their coordinated accumulation, whereas the negative correlations of green-note C6 alcohols (enriched in *D. rubrovalvata*) suggest divergent metabolic profiles between species (Fig. 7, Fig. 8A). Future transcriptomic and enzyme studies targeting phenylalanine ammonia lyase, terpene synthases, and acyltransferases would be needed to determine whether this co-occurrence is under transcriptional coordination and to identify genetic drivers of the more complex aroma and higher radical scavenging traits in *D. indusiata*.

### 4.6 Food chemistry implications and valorization potential

From a food chemistry perspective, *D. indusiata* cap is suggested to be a high-value functional ingredient, combining dense radical scavenging capacity (phenolic acids and phenylpropanoids) with a complex, intense aroma (terpenoid/ester bouquet, extremely high rOAVs for 1-octen-3-ol and α-ionone). These attributes make it attractive as a natural flavoring and antioxidant source for soups, sauces, plant-based meat analogues, and functional snacks. Conversely, *D. rubrovalvata* cap, with higher sugars and glutamate, and a greener, fattier aroma, is better suited for sweetness- and umami-driven applications, such as broths and seasonings, especially when combined with nucleotide-rich ingredients for umami synergy (Das et al., 2021; Kumar et al., 2021; Xia et al., 2025).

Several limitations should be acknowledged: First, only two *Dictyophora* species and two tissues were examined, and the sample size (*n* = 3) limits the ability to capture environmental or developmental variability. Second, sensory conclusions are inferred from rOAV and odor descriptors rather than validated by trained sensory panels. Third, the study is descriptive and does not directly interrogate the underlying genes or enzymes responsible for the observed metabolic differences. Future work should integrate transcriptomics and enzyme assays, assess bioaccessibility of key phenolics, conduct descriptive sensory analysis, and explore processing technologies (drying, fermentation) to preserve desirable traits. Collectively, this study provides a chemical basis for differentiating *Dictyophora* resources: *D. indusiata* cap offers higher radical scavenging capacity and suggested aroma complexity, while *D. rubrovalvata* cap complements with sweetness- and umami-oriented advantages.

## 5 Conclusion

This study provides an integrated, tissue-resolved flavoromic and metabolomic comparison of *D. indusiata* and *D. rubrovalvata*, revealing distinct chemotypes that explain their divergent sensory and functional properties. The *D. indusiata* cap emerged as a specialized metabolic hotspot, with total phenolics 1.6-fold higher (6.05 vs. 3.80 mg GAE/g DW), DPPH scavenging 3.3-fold higher (2.45 vs. 0.74 μmol TE/g DW), and ABTS scavenging 1.5-fold higher (4.85 vs. 3.15 μmol TE/g DW) than the *D. rubrovalvata* cap. Volatile analysis showed 2.1-fold higher total volatiles in *D. indusiata* cap (339.2 vs. 160.6 μg/g FW), with 497 up-regulated VOCs and 81 up-regulated phenolics. These quantitative differences were accompanied by a terpenoid/ester-rich aroma profile and elevated malic acid in the volva. In contrast, *D. rubrovalvata* cap had higher sugars and glutamic acid (4.56 vs. 2.13 mg/g DW), favoring sweetness and umami. The pronounced tissue- and species-specific divergence highlights the need for selective tissue utilization rather than whole-fruiting-body homogenization. Key limitations include the small sample size (*n* = 3), lack of sensory validation, and unknown phenolic bioavailability. Future work should integrate transcriptomics, sensory panels, and digestion models to confirm these chemotypes and support targeted valorization of *D. indusiata* cap as a high-value functional ingredient.

## CRediT authorship contribution statement

**Mingzheng Duan:** Writing – review & editing, Writing – original draft, Software, Resources, Methodology, Investigation, Funding acquisition. **Chenggang Zhao:** Writing – review & editing, Writing – original draft, Methodology, Investigation. **Qiuju Wu:** Writing – review & editing, Writing – original draft, Methodology, Investigation. **Ming Tao:** Writing – review & editing, Investigation. **Xue Wang:** Writing – review & editing, Investigation. **Tingfen Lu:** Writing – review & editing, Investigation. **Tingting Qu:** Writing – review & editing, Investigation. **Min Li:** Writing – review & editing, Investigation. **Ting Li:** Writing – review & editing, Investigation. **Lihua Tang:** Writing – review & editing, Investigation. **Muhammad Junaid Rao:** Writing – review & editing, Writing – original draft, Software, Investigation, Conceptualization. **Zeping Wang:** Writing – review & editing, Supervision, Resources, Funding acquisition, Conceptualization. **Xiaojian Wu:** Writing – review & editing, Supervision, Resources, Investigation, Funding acquisition, Conceptualization.

## Funding

This study was jointly funded by the 10.13039/501100001809National Natural Science Foundation of China (NNSFC 32560030, 32260715);Guangxi Science and Technology Program (GuiKe AA25069004)；Guangxi Special Crop Experimental Station (TS202223);Guangxi Academy of Agricultural Sciences Fund (GuiNongKe 2025YP065, GuiNongKe 2026YT171，GuiNongKe 2025YP076); Chinese Academy of Sciences Foresight Strategic Science and Technology Project (XDA0450302); Young Talent Project of Talent Support Program for the Development of Yunnan. Grant number: 210604199008271015. Guangxi Modern Agricultural Industry Technology System Innovation Team (gxxdnycyjstx2026-23-03).

## Declaration of competing interest

The authors declare that they have no known competing financial interests or personal relationships that could have appeared to influence the work reported in this paper.

## Data Availability

All data generated or analyzed during this study are included in this article and its supplementary files.

## Appendix A Supplementary data

Supplementary material 1 : Raw intensities and odor thresholds of volatile organic compounds detected in cap and volva tissues of *D. indusiata* (C) and *D. rubrovalvata* (H) by GC–MS.

Supplementary material 2 : Identification parameters of phenolic metabolites detected in cap and volva tissues of *D. indusiata* (C) and *D. rubrovalvata* (H) by UPLC-MS/MS. The table includes compound identifiers, mass spectrometric parameters, and identification levels.

Supplementary material 3 : OPLS-DA VIP scores and model parameters (R^2^X(cum), R^2^Y(cum), Q^2^(cum), etc.) of the volatile compounds detected in cap and volva tissues of *D. indusiata* and *D. rubrovalvata*.

Supplementary material 4 : Relative content (μg/g FW) of volatile organic compounds (VOCs) in each sample group.

Supplementary material 5 : Raw peak area values of volatile compounds used for hierarchical clustering (HCA) and principal component analysis (PCA).

Supplementary material 6 : Raw intensity values of volatile compounds used for k-means clustering.

Supplementary material 7 : Raw data for differential volatile compounds (filtered by |log₂FC| ≥ 1.0, VIP > 1.0, and *p* < 0.05) between *D. indusiata* cap (CCap) and *D. rubrovalvata* cap (HCap).

Supplementary material 8 : Odor descriptor classification and compound counts for flavor active profiling.

Supplementary material 9 : Pairwise correlation matrix among the top 50 discriminant volatile compounds (VIP > 1) between *D. indusiata cap* (CCap) and *D. rubrovalvata* cap (HCap).

Supplementary material 10 : Differential phenolic compounds between *D. indusiata cap* (CCap) and *D. rubrovalvata* cap (HCap).

Supplementary material 11 : Differential phenolic compounds between *D. indusiata* volva (CVol) and *D. rubrovalvata* volva (HVol).
